# Effects of Fertilizer Rates on Growth of *Brassica napus* L.

**DOI:** 10.3390/life16081351

**Published:** 2026-08-17

**Authors:** Yumei Xu, Yuqi Liu, Wenqing Tan, Guangyao Chen, Lei Qin, Yufang Li, Rongkui Hui

**Affiliations:** 1 Yuelushan Laboratory, Changsha 410128, China; 15760677702@163.com (Y.X.); 16673960921@163.com (Y.L.); twq8391290618@163.com (W.T.); qinlei@hunaas.cn (L.Q.); liyufang82@126.com (Y.L.); 2 College of Agriculture, Hunan Agricultural University, Changsha 410128, China; 3 Hunan Crop Research Institute, Changsha 410125, China; 4 Huazhi Bio-Tech Co., Ltd., Changsha 410125, China; chenguangyao@hitgentec.com

**Keywords:** *Brassica napus* L., yield, antioxidant enzyme activities, thiobarbituric acid reactive substances, transcriptomic analysis, *BnaC05g02370D*, *BnaC09g49910D*

## Abstract

The edible oil self-sufficiency rate of China is only approximately 30%, with rapeseed as the largest source of edible oil, contributing more than half of the total edible vegetable oil. Therefore, it is very important to achieve high rapeseed yield to ensure supply security. Fertilizer is one of the key factors affecting rapeseed yield and quality, and the appropriate use of fertilizer is essential for ensuring both high yield and high quality. To investigate the effects of fertilizer rates on the growth, yield, and quality of rapeseed, 18 rapeseed varieties were grown under two fertilizer input levels: normal fertilizer (NF, 15-15-15 NPK compound fertilizer at 600 kg·ha^−1^) and high fertilizer (HF, 15-15-15 NPK compound fertilizer at 1200 kg·ha^−1^). Agronomic traits, physiological indicators, yield, and quality of these varieties were systematically analyzed in this study. In addition, transcriptomic data from stems collected at the silique stage were used to identify key differentially expressed genes (DEGs), and the correlations between the expression levels of these candidate genes, physiological indicators, agronomic traits, and yield were examined. Compared with the NF treatment, the HF treatment promoted rapeseed growth during the bolting stage (except for root collar diameter). At harvest, 27.8% of the rapeseed varieties showed a significant increase in the number of effective branches per plant, while 33.3% exhibited significant increases in both the number of effective siliques per plant and the yield per plant. Among these varieties, Z01, Z02, Huayouza 50, and Huayouza 652 showed the greatest overall yield increases. The oil content decreased in 88.9% of the varieties, whereas the unsaturated fatty acid content increased in 77.8%, and the protein content increased in 94.4% of the varieties. Under HF treatment, superoxide dismutase activity in leaves at the bolting stage and peroxidase activity in leaves at the initial flowering stage both increased significantly (by 19.07% and 10.41%, respectively). The content of thiobarbituric acid-reactive substances (expressed as malondialdehyde equivalents) in stems at the initial flowering stage increased significantly (by 138.39%). In addition, catalase activity in leaves at the 3- to 4-leaf stage reached its highest value among all treatments (27.31 U·g^−1^·min^−1^·FW). The KEGG enrichment analysis indicated that the DEGs were significantly enriched in secondary metabolite biosynthesis, plant hormone signal transduction, plant–pathogen interactions, and metabolic pathways. Through screening against the NCBI database and subsequent validation by RT-qPCR, we ultimately identified four candidate DEGs: *BnaC05g02370D*, *BnaC09g49910D*, *BnaC03g45470D*, and *BnaA04g00130D*. Among these four varieties, catalase activity and malondialdehyde content in rapeseed stems at the initial flowering stage showed significant positive correlations with yield (r = 0.738 and r = 0.894, respectively). *BnaC09g49910D* maintained a consistently positive correlation with yield under both the HF and NF treatments (r = 0.977 and r = 0.806, respectively). In contrast, the direction of the association for *BnaC05g02370D* reversed depending on the fertilizer level (r = −0.858 under HF vs. r = 0.443 under NF). Catalase activity and the content of thiobarbituric acid-reactive substances in stems at the initial flowering stage and the expression levels of *BnaC05g02370D* and *BnaC09g49910D* in stems at the silique stage are associated with yield to varying degrees. These indicators can be used as candidate traits for assessing rapeseed yield potential under different fertilizer input levels.

## 1. Introduction

Rapeseed is one of the most important oilseed crops in China. In 2024, the national rapeseed planting area exceeded 7.9 million ha, with seed production surpassing 16.86 million metric tons. Rapeseed oil accounted for approximately 42% of the country’s total vegetable oil output. However, the edible oil self-sufficiency rate in China currently stands at only about 30%, indicating a persistently severe supply situation [1,2]. Against the backdrop of efforts to reduce fertilizer inputs while increasing use efficiency, identifying key genes that regulate the response of rapeseed to fertilizer and elucidating their association with yield and quality represents a crucial strategy for achieving higher yields with lower inputs. Fertilizer rates currently vary considerably across production regions. In nutrient-poor areas, inputs are insufficient, whereas in high-yielding regions, excessive fertilizer application is widespread [3]. Excessive fertilizer rates increase production costs, reduce nutrient use efficiency, and cause environmental pollution. It also predisposes rapeseed to lodging, resulting in yield losses [4]. Furthermore, the common practice of relying heavily on chemical fertilizers while neglecting organic fertilizer contributes to low fertilizer use efficiency, which in turn leads to soil degradation, nutrient imbalance, yield instability, and ecological risks [5]. Long-term excessive fertilizer application not only raises production costs but also exerts sustained pressure on the environment. Under intensive cropping systems, soil nutrients are substantially depleted, and the recovery period is short. Therefore, scientifically guided fertilization is essential for promoting rapeseed growth, enhancing nutrient uptake efficiency, increasing yield, and maintaining soil fertility [6,7]. Scientific and rational fertilization is directly associated with key yield components, including biological yield, number of seeds per silique, and thousand-seed weight, and also significantly influences seed protein and oil content [8]. Nitrogen, phosphorus, and potassium, as macronutrients, are involved in protein and nucleic acid synthesis, root development and energy metabolism, and oil synthesis and transport, respectively. They play critical roles in enhancing stress tolerance and lodging resistance and in improving yield [9,10,11]. When plants are subjected to nutrient stress, the balance of reactive oxygen species (ROS) is disrupted. When the stress intensity exceeds the cellular antioxidant capacity, excessive ROS accumulate and induce membrane lipid peroxidation, resulting in damage to the cell membrane structure and impairment of membrane function [12]. Zhang Qingxia et al. [13] used rapeseed to investigate the effects of deficiencies in different nitrogen forms on seedling growth, development, and physiological indicators and found that parameters such as POD activity, SOD activity, and MDA content exhibited significant changes. Cao Xianfu et al. [14] showed that under low-potassium stress, POD, SOD, and CAT activities, as well as MDA content, significantly increased in oats. These results indicated that ROS activity and MDA content (assessed via thiobarbituric acid-reactive substances, TBARS) can serve as sensitive response indicators of nutrient stress. Overexpression of *BnNRT2.3-like* enhanced the tolerance of rapeseed to low-nitrogen stress and increased yield [15]. The *BnaC01.CCT8* gene forms a protein complex with *BnARF18* to establish a regulatory network that promotes auxin synthesis and inhibits jasmonic acid synthesis, thereby significantly promoting silique elongation and seed development [16]. *BnRRF* encodes a ribosomal recycling factor; overexpression of this gene leads to larger seeds and increased seed weight in rapeseed [17]. Nitrogen nutrition indirectly regulates the expression of genes such as *BnRRF* by influencing ribosomal function and protein synthesis, thereby affecting seed development and yield. Regarding phosphorus regulation, candidate genes related to phosphorus homeostasis—such as *GPT1*, *MGD2*, and *SIZ1*—are co-localized with quantitative trait loci (QTLs) on chromosome A2 that regulate seed yield and number of seeds per silique. Changes in the expression of these genes under low-phosphorus conditions are significantly associated with yield traits [18]. Although these studies have made important progress at the level of gene function, current research on the effects of fertilizer on rapeseed yield and quality remains largely limited to descriptive analyses of agronomic traits or single indicators [19,20]. It remains unclear how fertilizer synergistically regulates physiological indicators and gene expression to influence yield and quality. The cross-level systemic mechanisms underlying this process also need to be elucidated.

In this study, 18 rapeseed varieties were used as experimental materials. Agronomic traits, physiological indicators, yield, and quality were analyzed under different fertilizer rates. By integrating transcriptomic data from stems collected during the silique stage, key differentially expressed genes were identified, and their associations with rapeseed growth and quality were explored, thereby providing a scientific basis for rational fertilization.

## 2. Materials and Methods

### 2.1. Experimental Materials

The experimental materials comprised 9 conventional rapeseed varieties: Zhongshuang 11, Z01, Z02, Z03, Zhongyou 814, Zhongyou 926, Zheyou 505, Zhongyou 827, and Shengyuan No. 1; and 9 hybrid *Brassica napus* varieties: Zhongyouza 652, Huayouza 50, Huayouza 652, Fengyou 789, Zheyouza 59, Zheyouza 313, Fengyou 958, Fengyou 823, and Fengyou 919. All materials were provided by the Yuelushan Laboratory in Hunan Province.

### 2.2. Experimental Design

The experiment was conducted from 2 October 2023 to 5 May 2024 at the Xunlonghe Rapeseed Experimental Base in Changsha City (28°20′ N, 113°12′ E), with rice as the preceding crop. The experimental field consisted of red soil with a uniform texture and flat topography, with no significant elevation differences among the plots. A randomized block design was employed, comprising two fertilizer levels and 18 varieties, resulting in a total of 36 treatment combinations. Fertilizer treatments included a high-fertilizer treatment (HF, 80 kg per 667 m^2^, equivalent to 1200 kg ha^−1^) and a conventional control (NF, 40 kg per 667 m^2^, equivalent to 600 kg ha^−1^) [21]. The fertilizer used was a 15-15-15 compound fertilizer. The application rates of pure nutrients in the HF treatment were 180 kg ha^−1^ each of N, P_2_O_5_, and K_2_O, while those in the NF treatment were 90 kg ha^−1^ each. Seeds were sown in rows, with plot areas of 5 m^2^ and a spacing of 25 cm within rows and 30 cm between rows. Before sowing, a basal application of compound fertilizer was made to each plot on 1 October 2023, at the dosage specified for each treatment. Weed control and pest and disease control were carried out uniformly during the seedling and bolting stages. All other field management practices were consistent across plots.

### 2.3. Experimental Methods

A total of 18 varieties were tested (see Section 2.1 for details). After yield measurement at harvest in May 2024, four varieties (Z01, Z02, Huayouza 50, and Huayouza 652) were selected based on their high baseline yield, their highest absolute yield under HF fertilizer, their synergistic increases in yield components, and an overall yield increase of more than 80%. These four varieties were then used for subsequent measurements of physiological parameters, quality analysis, and gene expression analysis. Among them, Z01 was subjected to transcriptomic sequencing, and the study of differentially expressed gene patterns was extended to all four varieties.

#### 2.3.1. Agronomic Traits Investigation

A pre-winter trait investigation was conducted on 12 January 2024, and harvest-season trait assessments were conducted on 5 May 2024. From each plot, five central plants with consistent growth and free of pests and diseases were randomly selected, and the mean value of each trait was recorded as the plot measurement.

Pre-winter seedling-stage indicators included the number of green leaves on the main stem, the total number of leaves on the main stem, maximum leaf length, maximum leaf width, root collar diameter, and plant height [22].

Harvest traits were plant height, effective branch height, effective length of the main inflorescence, number of primary effective branches, number of effective siliques per plant, root collar diameter, and yield per plant [23]. The detailed definitions of the trait indicators mentioned above are given in Supplementary Table S3.

Quality parameters were palmitic acid content, stearic acid content, oleic acid content, linoleic acid content, linolenic acid content, seed oil content, glucosinolate content, and protein content [24]. Measurements were performed using a near-infrared spectrometer (FOSS, Denmark, model NIRS DS2500, spectral range 850–2500 nm) in diffuse reflection mode, which directly scanned whole seeds without chemical pretreatment. The instrument was pre-programmed with global and regional calibrations, as well as built-in wavelength and optical standards, enabling stable measurements immediately upon use [25]. Furthermore, this method demonstrates good reproducibility in determining the oil content and fatty acid composition of rapeseed, with precision meeting national standards [26]. After harvesting seeds from the field plots, seed samples from the same variety and treatment were thoroughly mixed, reduced to equal volumes, filled into sample cups, and compacted before undergoing a single scan. The content was expressed as a mass fraction on a dry basis (%), and the resulting quality value reflected the average quality level of that variety under that treatment.

#### 2.3.2. Physiological Parameter Measurement

##### Sample Collection and Preparation

Leaf tissue samples were collected from rapeseed plants at the 3- to 4-leaf stage, bolting stage, and initial flowering stage, and lower stems were collected from rapeseed plants at the early flowering, full flowering, and silique stages. Immediately after sampling, the samples were placed in an ice box, refrigerated, and then stored in a −80 °C freezer for subsequent analysis. Fresh plant tissue (0.1 g) was weighed, 1.5 mL of 0.05 M phosphate buffer (pH 7.8) was added, and the mixture was ground in an ice bath. The homogenate was centrifuged at 4 °C (10,000× *g* for 20 min), and the supernatant was used as the enzyme solution. All experiments were performed following the techniques described by Xiao Langtao et al. [27].

##### SOD Activity Assay

The nitroblue tetrazolium (NBT) method of Giannopolitis et al. [28] was adopted. An aliquot (2 μL) of the enzyme solution was added to 300 μL of reaction buffer (containing Met, NBT, EDTA-Na_2_, MFD, etc.), exposed to light at 4000 lx for 30 s, and the absorbance was measured at 560 nm. Total SOD activity was expressed as absorbance per gram of fresh weight (U/g·FW) and calculated using the following formula, SOD activity = (A_CK_ − A_E_) × V/(W × 0.5 × A_CK×VE_). Three replicates were performed for each sample.

##### CAT Activity Assay

The ultraviolet absorption method of Beers et al. [29] was adopted. An aliquot (10 μL) of the enzyme solution was added to 250 μL of reaction mixture (containing 0.1 M H_2_O_2_ and 0.1 M phosphate buffer, pH 7.0), and readings were taken at 240 nm every 30 s for a total of five readings. CAT activity (U/g·FW/min) was calculated as follows: CAT activity = (A_min_ − A) × 1.5/(0.02 × 3 × W × 0.1). Three replicates were performed for each sample.

##### POD Activity Assay

The guaiacol method of Chance et al. [30] was adopted. An aliquot (2 μL) of the enzyme solution was added to 300 μL of reaction mixture (containing guaiacol and 30% H_2_O_2_), and readings were taken at 470 nm every 30 s for a total of five readings. POD activity (U/g·FW/min) was calculated as follows: POD activity = (A_2min_ − A_0_) × 1.5/(0.02 × W × 2).

##### TBARS Content Assay

The thiobarbituric acid (TBA) method of Heath et al. [31] and Hodges et al. [32] was adopted. The enzyme solution was mixed with 0.6% TBA solution at a 1:2 volume ratio. The tube was sealed and placed in a boiling water bath for 15 min. After rapid cooling, the mixture was centrifuged at 4000 rpm for 10 min, and the absorbance of the supernatant was measured at wavelengths of 450, 532, and 600 nm. TBARS content (μmol MDA eq.·g^−1^·FW) was calculated as follows: [6.45 (D_532_ − D_600_) − 0.56D_450_] × 0.015/W. Three replicates were performed for each sample. Results are expressed as TBARS in MDA equivalents.

#### 2.3.3. Transcriptome Screening and Expression Validation of Key Candidate Genes

##### Sample Collection and Preparation for Transcriptome Analysis

At the silique stage (9 April 2024), variety Z01, which differed significantly from the other varieties, was selected, and lower stem tissue was harvested, and three biological replicates were prepared. After sampling, the samples were immediately flash-frozen in liquid nitrogen and stored at −80 °C for later use. Total RNA was extracted using the RNApure Fast Plant Kit; genomic DNA was removed by DNase I digestion, and the RNA passed quality control.

##### Library Construction and Sequencing

Using 2 μg of total RNA, a strand-specific transcriptome library was constructed according to the instructions of the KC-Digital™ Stranded mRNA Library Prep Kit for Illumina^®^ (Wuhan Kangce Technology Co., Ltd., Wuhan, China, catalog number DR08502). This kit labels cDNA molecules with unique molecular identifiers (UMIs) consisting of 8 random bases to eliminate bias introduced by PCR amplification and sequencing replicates. The library was enriched for 200–500 bp fragments; after quantification, paired-end (PE150) sequencing was performed on a DNBSEQ-T7 sequencer.

##### Sequencing Data Processing

Raw sequencing data were first assessed using FastQC and MultiQC for quality control. Adapter sequences and low-quality reads were removed using fastp (v0.23.0) under the following criteria: reads containing more than five N bases, reads with more than 8% of bases having a quality score ≤ Q20, and reads shorter than 15 bp were discarded, yielding clean reads. Subsequently, a UMI deduplication script developed in-house by Kangce Technology was applied to cluster reads sharing identical UMI sequences. Within each cluster, pairwise alignments were performed to extract reads with >95% sequence identity, which were then grouped into subclusters. Multiple sequence alignment was conducted for each subcluster to generate a single consensus sequence, thereby eliminating errors and biases introduced by PCR amplification and sequencing. The deduplicated consensus sequences were then aligned to the *Brassica napus* reference genome ‘Double 11 (ZS11.v0)’ (NCBI BioProject: PRJNA293435) [33] using STAR (v2.5.3a). The alignment parameters were set as follows: -outSAMtype BAM SortedByCoordinate, -outReadsUnmapped Fastx, -readFilesCommand zcat, -outFilterMatchNminOverLread 0.66, -outSAMmapqUnique 60, -chimOutJunctionFormat 1, -chimSegmentMin 10, and -chimOutType SeparateSAMold. All other parameters were set to the default. Finally, featureCounts (Subread-1.5.1) was used to quantify the number of reads mapped to the exonic regions of each gene, generating a raw integer count matrix.

##### Differential Expression Analysis

Differential expression analysis was performed based on the raw integer count matrix using the edgeR package (v3.12.1). TMM normalization was performed using the calcNormFactors function, and low-expression genes with CPM < 1 across all samples were filtered out. Dispersion was estimated using estimateDisp and a design matrix ~0 + group, with group representing the fertilizer treatment (NF vs. HF), was constructed. Differentially expressed genes were identified using a quasi-likelihood F-test (glmQLFTest). Three biological replicates were included for each treatment. The screening threshold for differentially expressed genes was set at |log_2_(fold change)| > 1 and FDR < 0.05 (Benjamini–Hochberg correction). Based on the results of pathway enrichment analysis, the BnIR [34] rapeseed omics database and the NCBI database [35] were used to perform functional screening and retrieve sequences for differentially expressed genes in relevant pathways.

##### qPCR Validation

For the candidate genes identified through screening, specific primers were designed using the NCBI Primer-BLAST online tool [36] (accessed on 22 October 2024). The product length was set to 100–200 bp, the Tm value to 60 ± 2 °C, and the GC content to 40–60%. Primer specificity was verified via BLAST to ensure that the primers amplified only the target sequences. All primers were synthesized by Beijing Qingke Xinyi Biotechnology Co., Ltd. (Changsha, China), and their sequences are provided in Supplementary Table S4. Total RNA obtained from sequencing was used to synthesize cDNA, after which RT-qPCR validation was performed. The RT-qPCR reactions were conducted on a StepOne Plus instrument (Applied Biosystems, Waltham, MA, USA) under the following conditions: 1 cycle at 95 °C for 30 s; 40 cycles at 95 °C for 5 s and 60 °C for 30 s; and a melting curve analysis program ranging from 65 °C to 95 °C at a heating rate of 0.1 °C/s. BnActin was used as the internal control, and the relative expression levels of the target genes were calculated using the 2^−ΔΔCt^ method.

The RT-qPCR validation was carried out in two phases. In the first phase, Z01 was used as the material, with three independent biological replicates set for each treatment and three technical replicates per biological replicate, to validate differential gene expression. In the second phase, four varieties—Z01, Z02, Huayouza 50, and Huayouza 652—were selected; a single RNA sample from each variety and treatment was tested with three technical replicates per sample to investigate expression trends across varieties. The melting curves for all primers exhibited a single peak, indicating no non-specific amplification products.

#### 2.3.4. Analysis of Correlations with Key Fertility Traits

Following the methods of previous studies, Pearson correlation analysis was used to examine the associations between pre-winter traits [37,38], physiological indicators [39], and key gene expression levels [40] on the one hand and yield on the other. Significance levels were set at *p* < 0.05 and *p* < 0.01.

### 2.4. Statistical Data Analysis

Data organization and chart plotting were performed using WPS 2023 software. Two-way analysis of variance (ANOVA) and Pearson correlation analysis were conducted using IBM SPSS Statistics 24 software. Differentially expressed genes (DEGs) in the transcriptome were screened using TBtools-II (version 2.3) [41] and the Kangce Cloud Bioinformatics Platform (http://www.seqcloud.cc:8888/#/layout/index (accessed on 15 October 2024)).

## 3. Results

### 3.1. Effects of Different Fertilizer Rates on Agronomic Traits and Quality of Rapeseed

#### 3.1.1. Agronomic Traits of Pre-Winter Rapeseed Seedlings Under Different Fertilizer Rates

Two-way analysis of variance revealed that both variety and fertilizer treatment had significant main effects on most agronomic traits during the bolting stage, and the interaction between variety and fertilizer was also significant in most cases, as shown in Table 1. As shown in Table S5, compared with the control (NF treatment), 66.7% of rapeseed varieties under the high-fertilizer (HF) treatment exhibited an increase in the number of green leaves on the main stem. Of the varieties, 77.8% showed an increase in the total number of leaves on the main stem, 72.2% exhibited an increase in the length of the largest leaf, 61.1% showed an increase in the width of the largest leaf, 61.1% exhibited an increase in plant height, and 88.9% showed a decrease in root collar diameter. The mean number of green leaves on the main stem under the NF and HF treatments ranged from 6.6 to 9.4 and from 6.6 to 11.0, respectively. The mean root collar diameters were 1.38–2.26 cm under the NF treatment and 1.08–2.16 cm under the HF treatment. These preliminary results indicate that the HF treatment promoted the growth of some varieties during the rapeseed bolting stage, although the response differed markedly among varieties. At the same time, the HF treatment generally inhibited root collar diameter.

#### 3.1.2. Agronomic Traits of Rapeseed at Maturity Under Different Fertilizer Rates

Two-way ANOVA showed that both variety and fertilizer treatment had significant main effects on most agronomic traits, with the effects of fertilizer treatment on plant height and yield per plant being particularly pronounced, as F equaled 151.262 with *p* less than 0.001 for plant height and 50.125 with *p* less than 0.001 for yield per plant. The interaction effect between variety and fertilizer was also significant for most traits, as shown in Table 2. As shown in Table S6, the effects of the HF treatment on yield-related traits differed by variety. Compared with the control (NF treatment), 66.7% of rapeseed varieties under the HF treatment showed a significant increase in plant height (*p* < 0.05), 27.8% showed a significant increase in the number of effective branches per plant, 33.3% showed a significant increase in the number of effective siliques per plant, and 33.3% showed a significant increase in yield per plant. Further pairwise comparisons of yield per plant between the NF and HF treatments within each variety showed that four varieties, namely Z01, Z02, Huayouza 50, and Huayouza 652, exhibited a significantly higher yield per plant under the HF treatment than under the NF treatment, with *p* less than 0.01, and the yield increases were particularly notable. These four varieties already possessed a high yield potential under the NF treatment. After the HF treatment, their yield per plant reached a high level among the tested materials, and their yield-determining factors, namely the number of effective siliques per plant and the number of first-order effective branches, increased simultaneously, indicating a robust yield-enhancing effect. Although the interaction effect between variety and fertilizer on yield per plant was not significant, with *p* equal to 0.138, this does not contradict the findings of the within-variety pairwise comparisons, because the two analyses reflect different perspectives.

#### 3.1.3. Rapeseed Quality Indicators Under Different Fertilizer Rates

As shown in Table S7, 88.9% of the varieties under the HF treatment exhibited a decrease in oil content, and 61.1% showed a decrease in saturated fatty acid content and glucosinolate content, compared with the NF treatment. In contrast, 77.8% of the varieties showed an increase in unsaturated fatty acid content, and 94.4% showed an increase in protein content. These results indicate that the HF treatment increased the unsaturated fatty acid and protein contents but reduced the oil content, saturated fatty acid content, and glucosinolate content in rapeseed [42]. Together with the results in Table 2, these findings suggest that the HF treatment promoted yield increases in some varieties while reducing the oil content in the vast majority of varieties. Therefore, appropriate fertilization is critical for rapeseed quality development, and fertilizer rates should be determined based on the characteristics of the cultivated varieties and the production objectives.

### 3.2. Effects of Different Fertilizer Rates on Physiological Indicators of Rapeseed

**Figure 1:**
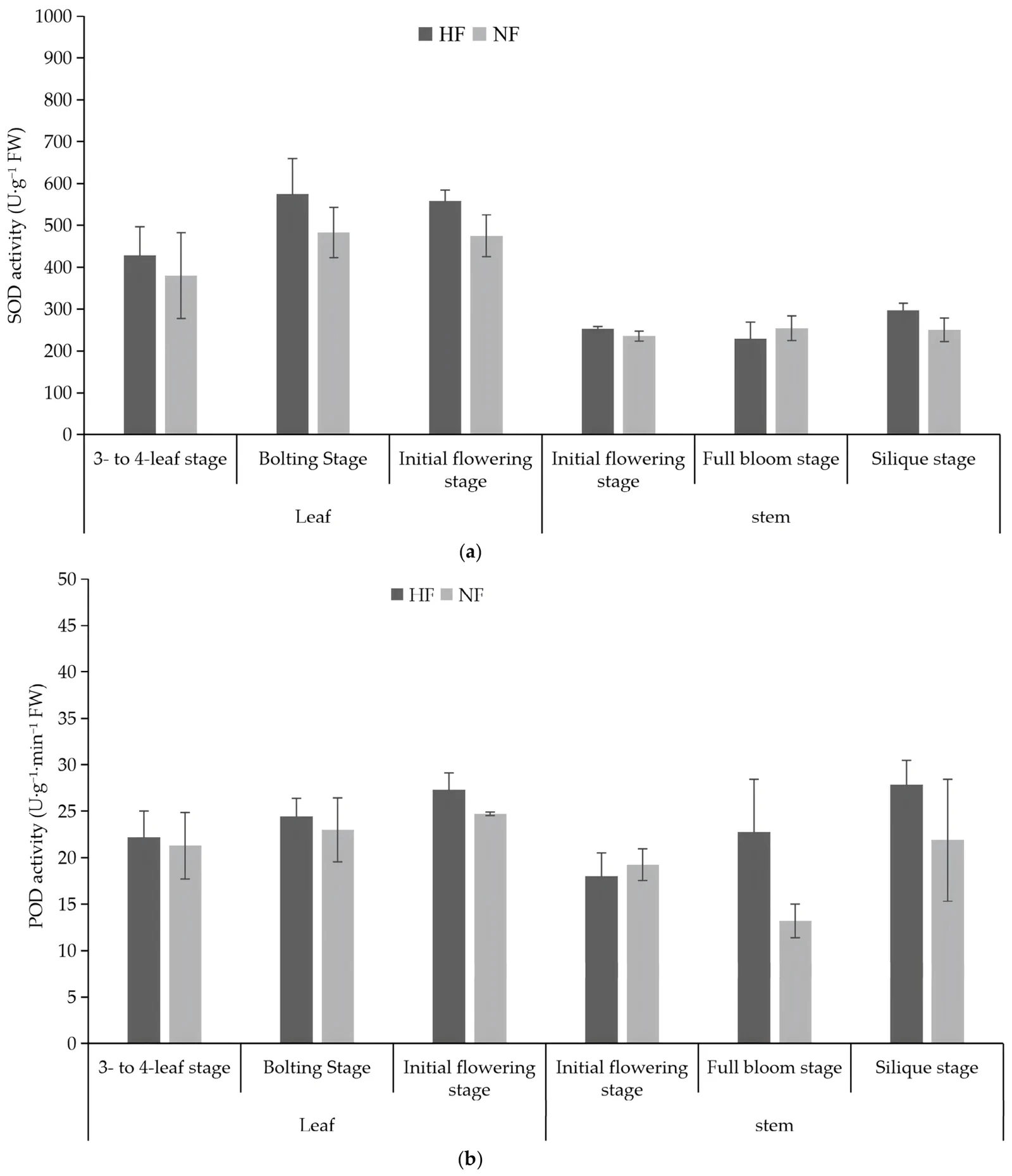

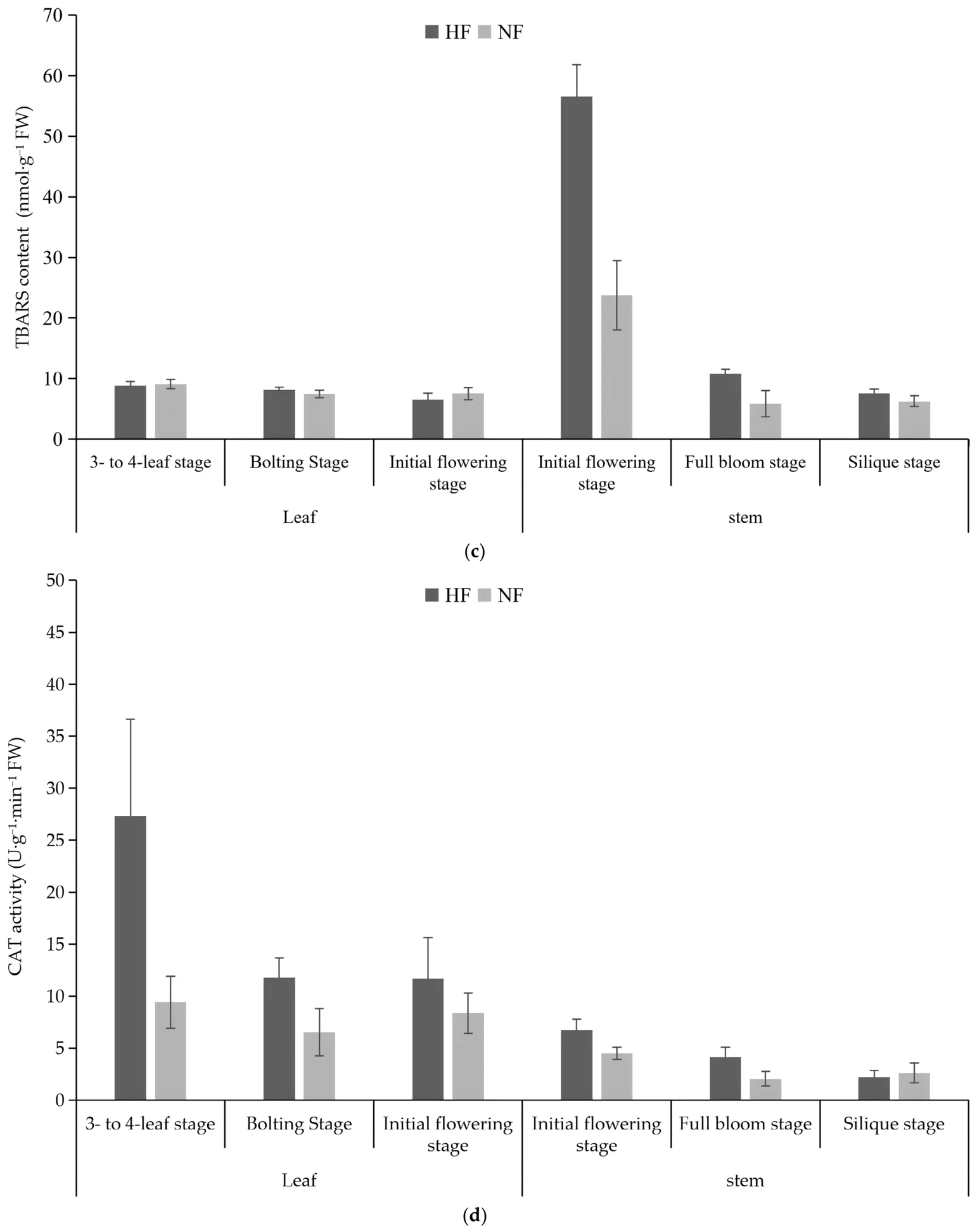
Contents of physiological indexes under different fertilizer rates. (**a**) SOD activity. (**b**) POD activity. (**c**) TBARS content. (**d**) CAT activity. Based on the yield performance of each variety under different fertilizer application rates, four varieties, namely ‘Z01’, ‘Z02’, ‘Huayouza 50’, and ‘Huayouza 652’, were selected for physiological parameter measurements. These varieties were chosen for their relatively high baseline yields, highest absolute yields under high-fertility conditions, and synergistic improvements in yield components. Leaf samples were collected at the 3- to 4-leaf stage, bolting stage, and initial flowering stage, while stem samples were collected at the initial flowering stage, full bloom stage, and silique stage. SOD, POD, and CAT activities, as well as TBARS content, were measured. With the variety serving as the replicate (*n* = 4), differences in physiological indicators at each stage between the HF and NF treatments were compared. Due to the limited sample size, this study focused primarily on describing trends without reporting significance levels. According to the Wilcoxon test, the differences between the two treatments at all stages were not statistically significant (*p* > 0.05).

As shown in Figure 1, significant differences were observed in SOD, POD, and CAT activities, as well as TBARS content, among the different fertilizer treatments. Under the HF treatment, leaf SOD activity was 12.72%, 19.07%, and 17.54% higher than that under the NF treatment at the 3- to 4-leaf stage, bolting stage, and initial flowering stage, respectively. Stem SOD activity was 7.34% and 18.56% higher at the initial flowering stage and silique stage, respectively, but 10.01% lower at the full bloom stage. Leaf POD activity was 6.35% and 10.41% higher at the bolting stage and initial flowering stage, respectively. Stem POD activity was 6.59% lower at the initial flowering stage but 72.58% and 27.36% higher at the full bloom stage and silique stage, respectively. Leaf CAT activity was 190.13%, 80.61%, and 39.62% higher at the 3- to 4-leaf stage, bolting stage, and initial flowering stage, respectively, and peaked at the 3- to 4-leaf stage (27.31 U/g·FW). Stem CAT activity was 50.00% and 100.49% higher at the initial flowering stage and full bloom stage, respectively, but 14.45% lower at the silique stage. Leaf TBARS content was 9.29% higher at the bolting stage but 2.76% and 13.01% lower at the 3- to 4-leaf stage and initial flowering stage, respectively. Stem TBARS content showed a declining trend over time but remained 138.39%, 84.30%, and 20.73% higher than that under the NF treatment at the initial flowering stage, full bloom stage, and silique stage, respectively. These results indicate that increasing fertilizer application significantly enhanced the activities of SOD, POD, and CAT [43,44], while TBARS content also showed pronounced accumulation in the stem at the early flowering stage.

### 3.3. Screening Key Genes Affecting Stem Growth of Brassica napus Under Different Fertilizer Rates

The silique stage is a critical period for seed filling and the accumulation of oil and protein in rapeseed [45]. During this stage, gene expression patterns in the stem—the primary transport pathway for assimilates-directly regulate the efficiency of nutrient allocation to the siliques.

#### 3.3.1. Transcriptome Sequencing Quality Statistics and Functional Enrichment Analysis

##### Quality Statistics

As shown in Table 3, for all samples, Q20 ranged from 98.22% to 98.36%, Q30 ranged from 95.82% to 96.07%, and the GC content ranged from 46.96% to 47.21%. The effective data proportion ranged from 89.60% to 90.61%, the total mapping rate ranged from 97.92% to 98.22%, and the unique mapping rate ranged from 93.90% to 94.45%. These results indicate that the sequencing data quality is reliable and can be used for subsequent differential expression analysis [46].

##### Principal Component Analysis (PCA)

**Figure 2:**
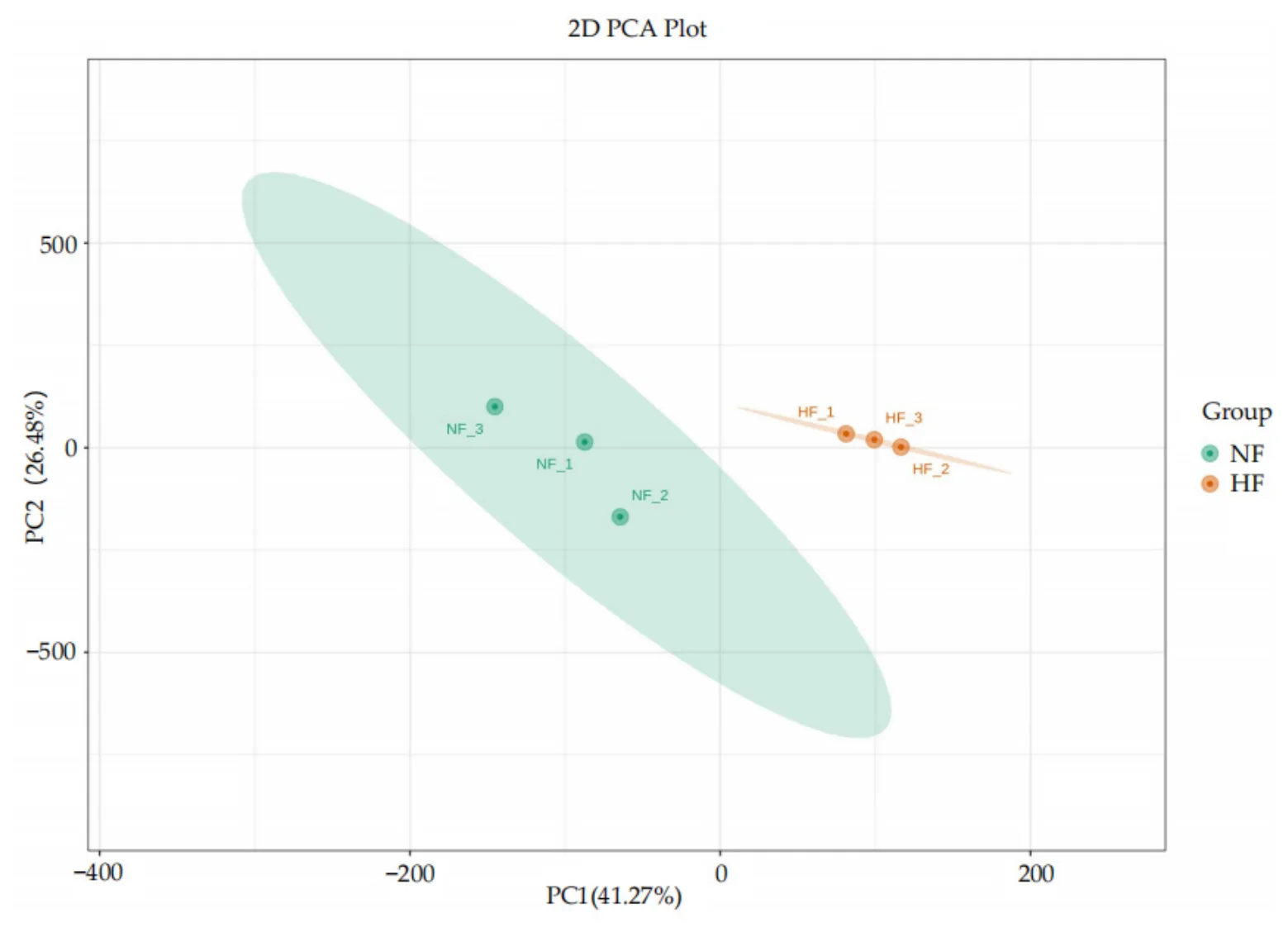
Principal component analysis (PCA) of transcriptomes. PCA was performed based on log2-transformed RPKM values of all expressed genes in the NF (blue, *n* = 3) and HF (red, *n* = 3) groups.

Based on the log2-transformed RPKM values of all genes, principal component analysis (PCA) was performed to evaluate the overall transcriptomic divergence between the NF and HF groups. The first two principal components (PC1 and PC2) accounted for 41.27% and 26.48% of the total variance, respectively. As shown in Figure 2, the three biological replicates within each group clustered tightly together, indicating high reproducibility. A clear separation between the NF and HF groups was observed along PC1, suggesting that the increased application rate of compound fertilizer was associated with a significant alteration of the global transcriptomic landscape.

##### Expression Analysis of Differentially Expressed Genes

Using edgeR [47], a total of 4453 differentially expressed genes (DEGs) were identified, including 2406 upregulated and 2047 downregulated genes.

##### GO Classification and Enrichment Analysis of Differentially Expressed Genes

**Figure 3:**
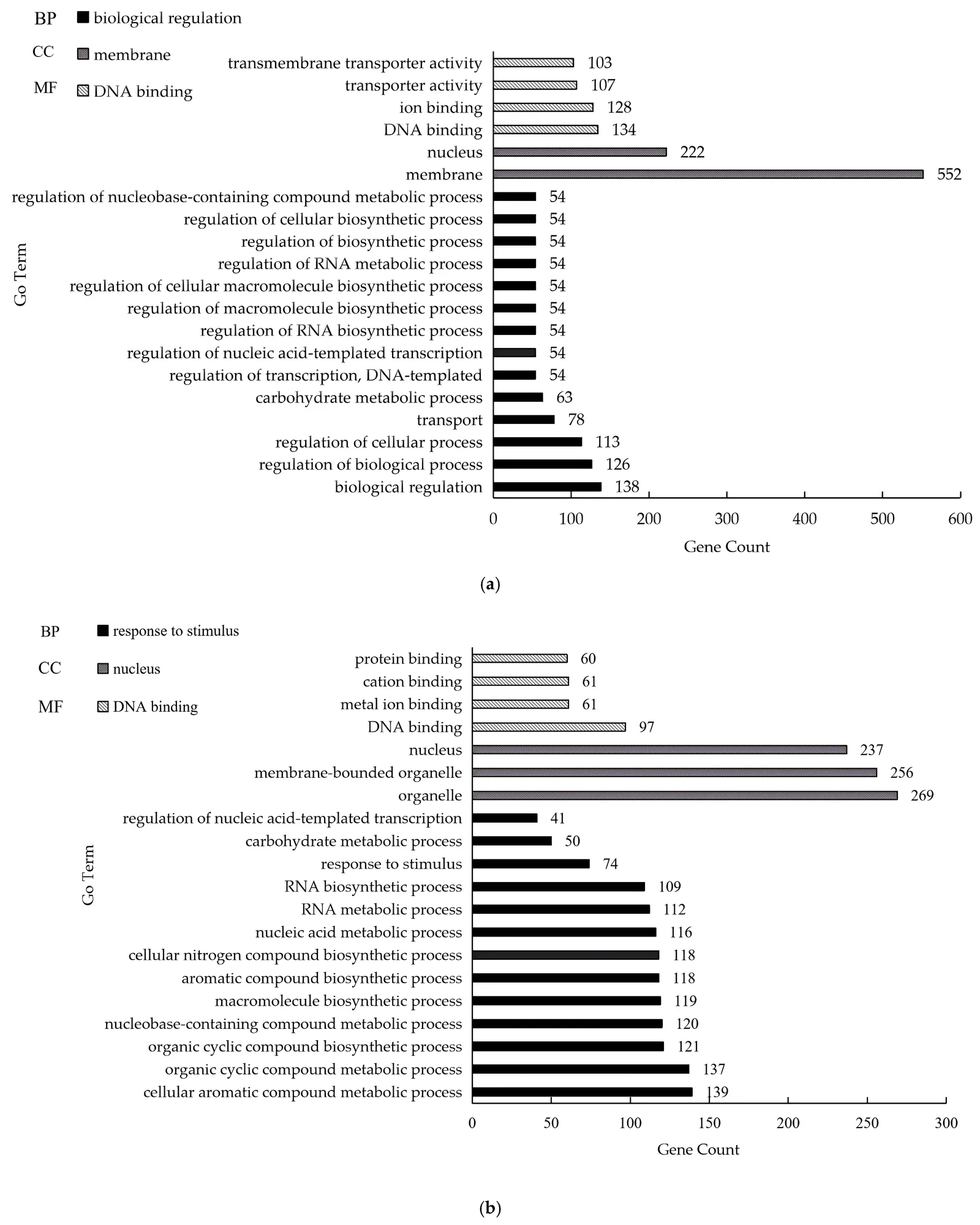
GO enrichment analysis of differentially expressed genes. (**a**) Top 20 significantly enriched GO terms in upregulated genes. (**b**) Top 20 significantly enriched GO terms in downregulated genes. BP: biological process; CC: cellular component; MF: molecular function. The screening threshold was set at *p* < 0.05 and FDR < 0.05. The same threshold was applied to the figure below.

The GO functional enrichment analysis (Figure 3) classified the differentially expressed genes into three major categories: biological process (BP), molecular function (MF), and cellular component (CC). Overall, the differentially expressed genes were most significantly enriched in the cellular component category. Upregulated genes (Group A) were significantly enriched in terms such as membrane, transporter activity, and ion binding, whereas downregulated genes (Group B) were primarily enriched in terms related to nucleus and DNA binding and were broadly involved in biological processes including nucleobase-containing compound metabolic process and regulation of transcription, DNA-templated.

##### KEGG Pathway Enrichment Analysis of Differentially Expressed Genes

**Figure 4:**
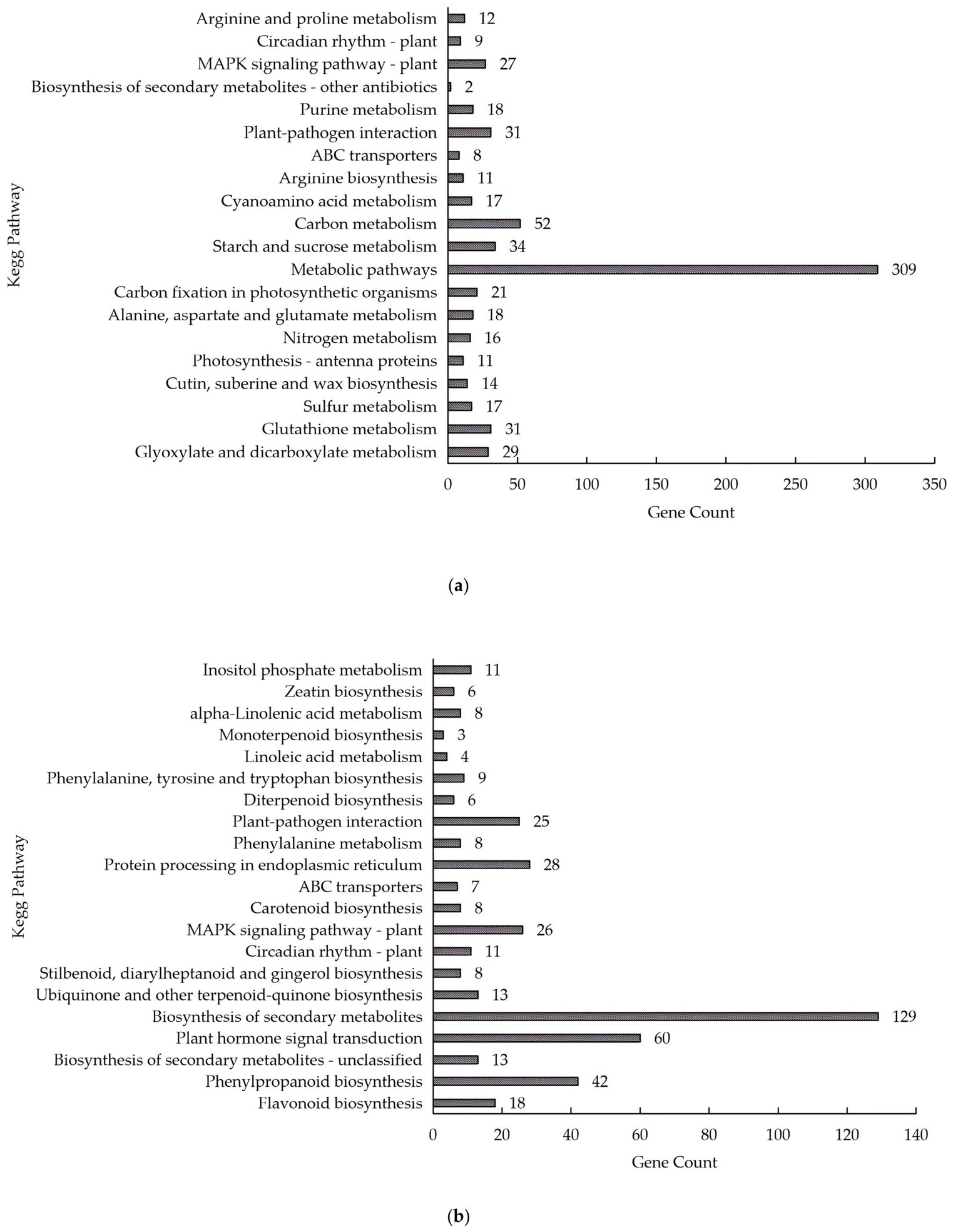
KEGG enrichment analysis of differentially expressed genes. (**a**) Top 20 significantly enriched KEGG pathways in upregulated genes. (**b**) Top 20 significantly enriched KEGG pathways in downregulated genes.

KEGG enrichment analysis (Figure 4) showed that the differentially expressed genes were significantly enriched in pathways such as biosynthesis of secondary metabolites, plant hormone signal transduction, phenylpropanoid biosynthesis, plant–pathogen interaction, and metabolic pathways. Among these, upregulated genes were primarily enriched in primary metabolic pathways, including metabolic pathways, carbon metabolism, and nitrogen metabolism, whereas downregulated genes were concentrated in the plant hormone signal transduction and biosynthesis of secondary metabolites pathways.

#### 3.3.2. Validation of Transcriptome Sequencing Results and Key Gene Screening

**Figure 5:**
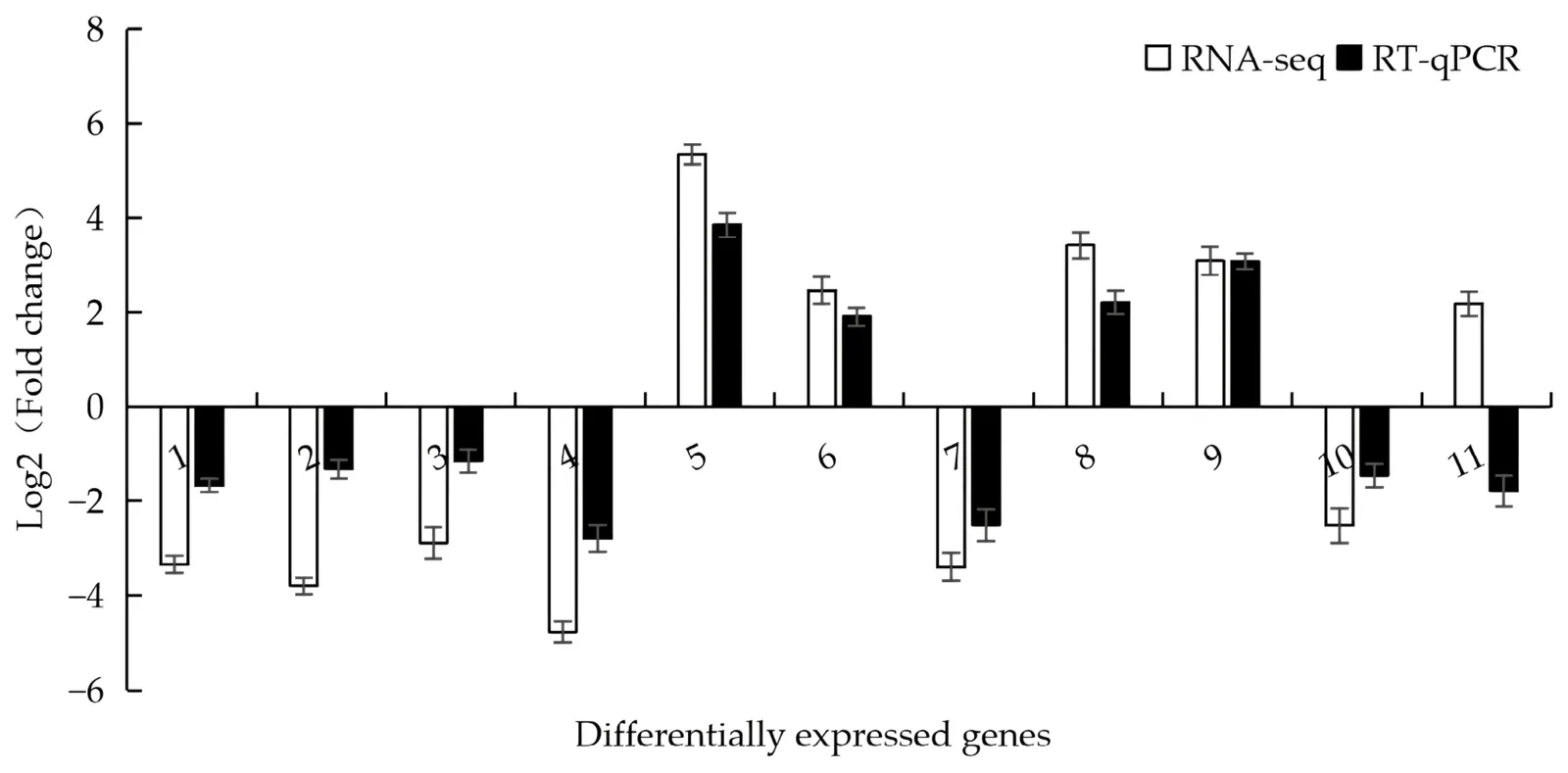
RT-qPCR validation of the expression results of the transcriptome data. Numbers (1–11) correspond to the following gene IDs: 1, *BnaC08g09640D*. 2, *BnaA10g02490D*. 3, *BnaA03g36950D*. 4, *BnaC05g02370D*. 5, *BnaA06g02010D*. 6, *BnaC08g19010D*. 7, *BnaC09g49910D*. 8, *BnaC04g01830D*. 9, *BnaC03g45470D*. 10, *BnaA04g00130D*. 11, *BnaA08g24500D*. RNA extracted from silique-stage stems (provided by the company) was used for RT-qPCR validation. Pearson correlation analysis showed r = 0.879, R 2 = 0.772, *p* < 0.001.

To validate the accuracy of the sequencing results and identify candidate genes associated with the effects of fertilizer on the growth of rapeseed, 11 candidate differentially expressed genes from the Z01 variety, which are involved in metabolic pathways such as plant hormone signal transduction, biosynthesis of secondary metabolites, and nitrogen metabolism, were selected for RT-qPCR validation (Figure 5). *BnActin* was used as the internal reference gene. The RT-qPCR primers for the differentially expressed genes are listed in Table 1. Relative gene expression levels were calculated using the exponential function method [48]. The RT-qPCR validation results were 90.9% consistent with the sequencing results, indicating that the expression data obtained from sequencing were reliable.

#### 3.3.3. Analysis of Expression Trends of Key Differentially Expressed Genes Across Different Varieties

**Figure 6:**
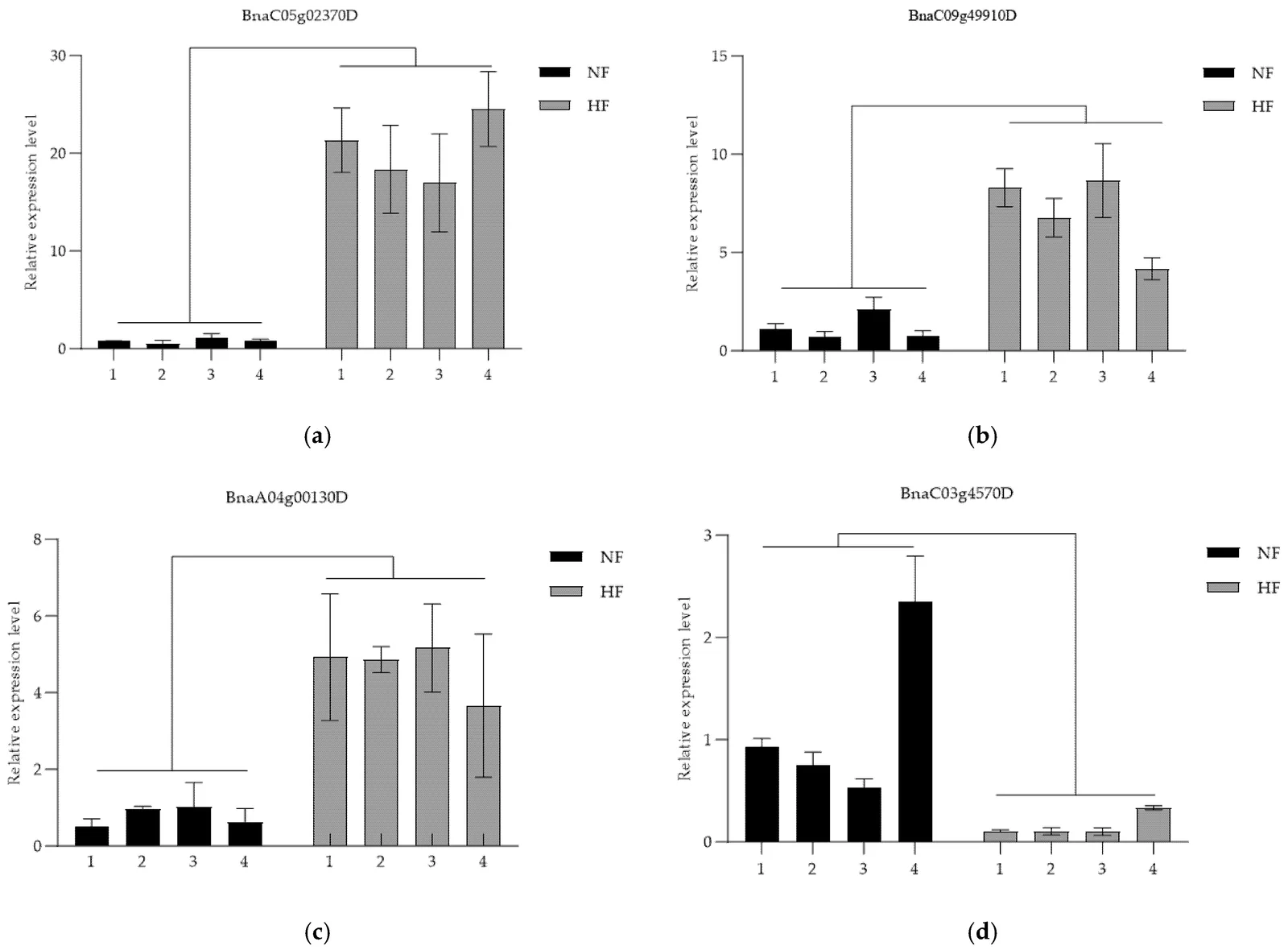
Gene expression under different fertilizer rates. (**a**) *BnaC05g02370D*. (**b**) *BnaC09g49910D*. (**c**) *BnaA04g00130D*. (**d**) *BnaC03g45470D*. Using Z01, Z02, Huayouza 50, and Huayouza 652 grown under different fertilizer application rates as materials, stems were collected at the silique stage, and the relative expression levels of *BnaC05g02370D*, *BnaC09g49910D*, *BnaC03g45470D*, and *BnaA04g00130D* were determined. For each variety and each treatment, a single sampling measurement was performed, which was used only to observe the expression response trends of the four genes to fertilizer application in varieties with different genetic backgrounds; therefore, no statistical comparison was conducted between varieties or between treatments.

Based on the above RT-qPCR validation results, combined with bioinformatics analysis and comparison with the NCBI database, four key differentially expressed genes, namely *IAA17*, *PYL5*, *PR1*, and *IPT3*, were identified from the 11 candidate differentially expressed genes. To further investigate the response patterns of these genes to fertilizer application in varieties with different genetic backgrounds, four varieties, Z01, Z02, Huayouza 50, and Huayouza 652, were selected. RT-qPCR was used to detect the expression levels of the four genes in the stems during the silique stage under NF and HF treatments, and the results are shown in Figure 6. Among the four varieties tested, the relative expression levels of *IAA17*, *PYL5*, and *IPT3* under the HF treatment were generally higher than those under the NF treatment, whereas the opposite was observed for *PR1*. The four varieties exhibited relatively consistent upward and downward trends, suggesting that this fertilizer-response regulatory pattern may be conserved to some extent across different varieties of Brassica napus. However, this preliminary trend requires further validation through experiments involving multiple varieties and multiple replicates.

### 3.4. Correlation Analysis of Rapeseed Yield with Key Gene Expression, Physiological Indicators, and Pre-Winter Growth Traits

Based on yield performance, this study selected four rapeseed varieties that exhibited a high baseline yield under normal fertilizer application, the highest absolute yield under high-fertilizer application, synergistic improvements in yield components, and a yield increase exceeding 80%. Using the method described in Section 2.3.4, this study conducted an association analysis between yield and the expression of key genes, physiological indicators, and pre-winter growth traits, and preliminarily examined the association patterns between yield and physiological, molecular, and agronomic traits under different fertilizer responses. In this section, the sample size for each treatment was four varieties (*n* = 4), and the results were used for the exploratory association evaluation of candidate indicators.

#### 3.4.1. Correlation Analysis of Pre-Winter Traits and Yield in Rapeseed

A correlation analysis was performed between rapeseed pre-winter traits and yield (Table 4). Under the NF treatment, leaf width showed a significant positive correlation with yield (r = 0.961, *p* < 0.05). Under the HF treatment, the total number of leaves on the main stem (r = −0.762) and plant height (r = −0.535) were negatively correlated with yield, indicating that, under high-fertilizer application, certain vegetative growth traits were negatively associated with yield. It is worth noting that, although both the total number of leaves on the main stem and plant height were negatively correlated with yield under the HF treatment, the average yield under the HF treatment remained higher than that under the NF treatment. Thus, no consistent positive correlation pattern was observed between these two traits and the yield increase.

#### 3.4.2. Correlation Analysis Between Physiological Indicator Levels and Rapeseed Yield

A correlation analysis was conducted between physiological parameters and rapeseed yield (Table 5). In rapeseed stems, CAT activity at the initial flowering stage (r = 0.738, *p* < 0.05) and at the full flowering stage (r = 0.753, *p* < 0.05) was significantly and positively correlated with yield. TBARS content at the initial flowering stage showed a highly significant positive correlation with yield (r = 0.832, *p* < 0.01). The POD content at the full flowering stage also showed a highly significant positive correlation with yield (r = 0.832, *p* < 0.01). These results indicate that, among the above four varieties, CAT activity and TBARS content in rapeseed stems during the initial flowering stage were significantly positively correlated with yield, suggesting that these two parameters may be associated with rapeseed yield potential and can serve as candidate physiological indicators for further verification.

#### 3.4.3. Correlation Analysis Between the Gene Expression Levels in Rapeseed and Yield

A correlation analysis was conducted between the expression levels of differentially expressed genes and yield (Table 6). At the silique stage of rapeseed, the expression level of *PYL5* in the stem showed a highly significant positive correlation with yield under the HF treatment (r = 0.977, *p* < 0.05) and a positive correlation under the NF treatment (r = 0.806). In contrast, *IAA17* showed a negative correlation with yield under the HF treatment (r = 0.806) but a positive correlation under the NF treatment (r = 0.443). These correlation patterns indicate that, among the above four varieties, different genes may have distinct underlying associations with yield, and that the relationship between the expression patterns of *PYL5* and *IAA17* and yield differs under different fertilizer regimes. Therefore, *PYL5* and *IAA17* can be preliminarily regarded as candidate genes associated with the yield response.

## 4. Discussion

### 4.1. The Effects of Different Fertilizer Rates on Rapeseed Yield, Quality, and Fertilizer Costs

Rapeseed is a crop with high nutrient requirements, and a relatively large amount of fertilizer must be applied to achieve high yields. Traditional high-yield cultivation practices typically involve applying nitrogen, phosphorus, and potassium fertilizers in multiple applications—including basal fertilizer, seedling fertilizer, pre-winter fertilizer, and bolting-stage fertilizer—to meet the nutrient demands of each growth stage and ensure stable and high yields. In recent years, due to a decline in the rural labor force, the frequency of rapeseed fertilization has decreased, resulting in excessive single-application rates. This has led to fertilizer leaching, low utilization efficiency, and consequently, high costs and low economic returns [49]. Therefore, rational fertilization is key to improving soil fertility, enhancing soil productivity, and achieving sustained high and stable yields. Increasing the fertilizer rate helps increase the number of green leaves, maximum leaf length, and leaf width of rapeseed before winter. Scientific fertilization not only facilitates safe overwintering but also effectively enhances yield [50,51,52]. Under the HF treatment, the number of green leaves on the main stem, total number of leaves, maximum leaf length, leaf width, and plant height at the bolting stage were all higher than those under the NF treatment, while root collar diameter decreased. This indicates that the HF treatment promoted plant growth and development at the bolting stage. This is consistent with previous findings showing that increased nitrogen fertilizer significantly increases the number of primary effective branches [53] and significantly improves key yield components such as plant height, number of effective siliques per plant, number of seeds per silique, and thousand-seed weight [54]. In the present study, the HF treatment resulted in improvements in plant height, number of primary effective branches, number of effective siliques per plant, root collar diameter, and yield per plant compared with the NF treatment, further corroborating these findings. However, it is worth noting that the promoting effect of the HF treatment on the aforementioned yield-related traits was observed only in certain varieties, indicating a clear genotype-dependent response. However, while the HF treatment increases seed yield, it is also accompanied by a decrease in oil and glucosinolate contents and an increase in protein content [55,56], which enhances the feed value of the seeds. Gao Jianqin et al. [57] reported that the nitrogen application rate was highly significantly negatively correlated with oil content and highly significantly positively correlated with protein content, which is consistent with the findings of Wu Yongcheng et al. [58]. Therefore, although the HF treatment increased seed yield, it did so at the expense of oil content, highlighting the need to coordinate yield and quality in rapeseed production. Meanwhile, the high-fertilizer treatment also carried risks of lodging-induced yield losses and increased costs. Previous research [19] has shown that the input–output ratio tends to decline once the optimal economic fertilizer application rate is exceeded, indicating that blindly pursuing maximum yield is not economically viable. In the present experiment, 88.9% of the varieties produced lower yields under the NF treatment than under the HF treatment. However, the risks of lodging and increased costs must also be weighed. Therefore, while pursuing high yields, it is necessary to comprehensively consider the balance among lodging risk, fertilizer costs, and yield gains.

### 4.2. Correlations Between Stress Resistance Indicators and Key Genes and Yield Under Different Fertilizer Treatments, and Their Value for Early Diagnosis

In addition to regulating rapeseed growth and yield, fertilizer application is also associated with changes in physiological and metabolic processes. Appropriate nitrogen application enhances SOD and POD activities in crop leaves [59], and pre-winter fertilizer has also been shown to increase SOD and CAT activities in rapeseed [60]. In the present study, the SOD, POD, and CAT activities under the HF treatment were higher than those in the control. Although enzyme activities are susceptible to sampling conditions and operational factors, systematic errors were effectively controlled in this study by standardizing the sampling site, using liquid nitrogen for rapid freezing, and performing three biological replicates. This ensured that the relative differences in enzyme activity among the treatments were comparable, suggesting that reactive oxygen species accumulation may be exacerbated under high-fertilizer conditions, thereby inducing a response from the antioxidant system. It is worth noting that the sharp increase in stem TBARS content during the initial flowering stage coincides with the critical period when rapeseed transitions from vegetative to reproductive growth, and functional impairment of the stems during this period may pose a potential threat to subsequent yield formation. Therefore, the stem TBARS level during the initial flowering stage can be preliminarily considered a candidate indicator for assessing the response to fertilizer stress, while the relationship between oxidative damage and final yield remains to be further discussed and verified.

These physiological changes were further corroborated at the transcriptional level. GO enrichment analysis revealed that the upregulated genes (Group A) were significantly enriched in categories such as membrane, transporter activity, and ion binding, whereas the downregulated genes (Group B) were primarily enriched in categories related to the nucleus and DNA binding and were broadly involved in biological processes including the nucleobase-containing compound metabolic process and the regulation of transcription, DNA-templated. Under HF treatment, rapeseed may respond to nutrient stress by enhancing membrane transport and ion transport functions while globally suppressing growth-related transcription and biosynthetic activities, together reflecting an adaptive trait characterized by accelerated uptake and slowed growth. KEGG analysis further identified three significantly enriched pathways closely associated with fertilizer response and nutrient allocation: plant hormone signal transduction, biosynthesis of secondary metabolites, and nitrogen metabolism. The plant hormone signal transduction pathway encompasses multiple signaling branches, including IAA, ABA, and CTK, all of which play key roles in plant responses to nutrient stress and the regulation of growth and development. Hao et al. [61] also reported that the differentially expressed genes within this pathway were significantly enriched across several nitrogen-level comparison groups in their transcriptomic analysis of rapeseed development under different nitrogen application rates. This finding is consistent with the results of the present study. In this study, the candidate genes *IAA17* and *PYL5*, which belong to this pathway, showed an upregulated trend under the HF treatment, whereas *PR1* showed a downregulated trend. *IAA17* is a negative regulator of auxin signaling and is involved in root architecture regulation [62,63]. *PYL5* encodes an ABA receptor that participates in stress responses by inhibiting SnRK2 kinase activity [64]. *PR1*, a marker gene of the salicylic acid pathway, is involved in plant defense responses [65]. This suggests that HF treatment may be associated with changes in the activity of the auxin, ABA, and salicylic acid signaling branches in the stem. The biosynthesis of secondary metabolites pathway includes subpathways such as phenylpropanoid metabolism and flavonoid synthesis. Within this pathway, the synthesis of lignin monomers in the phenylpropanoid pathway directly affects stem mechanical strength, whereas flavonoids are closely related to the antioxidant capacity of plants. Hao et al. [61] further found that the phenylpropanoid biosynthesis pathway, a component of this pathway, was significantly enriched, corroborating the results of this study and suggesting a possible association between nitrogen fertilizer application levels and the activity of this pathway. The candidate gene *IPT3*, which belongs to this pathway, encodes a key enzyme for CTK synthesis. It is regulated by nitrate signaling and is involved in modulating the root–shoot growth balance and the number of branches [66,67]. In this study, IPT3 showed an upregulated trend under the HF treatment. The nitrogen metabolism pathway may be involved in the plant’s absorption, assimilation, and redistribution of nitrogen. In the present study, this pathway was significantly enriched, which is consistent with the transcriptomic analysis of rice tillering buds conducted by Huang Xingyu [68]. That study found that differentially expressed genes under different nitrogen concentrations were highly significantly enriched in the nitrogen metabolism pathway, and that all of these genes encoded nitrate reductase-related proteins. The quality changes observed under the HF treatment in this study, namely a decrease in oil content and an increase in protein content, are hypothesized to be related to changes in the activity of the nitrogen metabolism pathway. When nitrogen supply is adequate, more carbon skeletons are utilized for amino acid and protein synthesis, which may reduce the availability of substrates for fatty acid synthesis; this reduced availability is further hypothesized to be associated with the decrease in oil content. However, this mechanism has not yet been directly verified within the scope of this study.

By integrating agronomic traits, physiological and biochemical indicators, and transcriptomic data, this study conducted a preliminary comprehensive analysis at the molecular and physiological levels, building on previous research that primarily focused on the apparent effects of fertilization on rapeseed yield and quality [69,70]. Correlation analysis revealed that CAT activity at the 3- to 4-leaf stage and at the initial flowering stage, POD activity at the peak flowering stage, and TBARS content at the initial flowering stage were all positively correlated with individual plant yield (r = 0.903, 0.738, 0.832, and 0.894), further supporting the possibility of a close association between rapeseed stress tolerance and final yield. *PYL5* showed a consistent positive correlation with yield under both HF and NF treatments (r = 0.977 under HF, *p* < 0.05; r = 0.806 under NF), whereas *IAA17* exhibited a negative correlation with yield under HF (r = −0.858) that shifted to a positive correlation under NF (r = 0.443), indicating that the direction of the relationship between expression levels and yield changed markedly with fertilizer application level. These results indicate that *PYL5* and *IAA17* are candidate genes associated with yield response. The positive correlations between the aforementioned physiological indicators and yield, as well as the associations between *PYL5*, *IAA17*, and yield, are based on correlation analyses across four varieties (*n* = 4); the identification of these candidate genes originated from the Z01 transcriptome, and their cross-variety expression patterns have been consistently validated across the four varieties. The study found that, across the four varieties, the expression levels of key genes such as *PYL5* and *IAA17* in the stems during the silique stage, as well as indicators such as CAT activity and TBARS content at the initial flowering stage, all showed preliminary associations with yield and can serve as candidate indicators for further validation.

### 4.3. Limitations and Perspectives

It should be noted that although this study provides a preliminary basis for screening candidate indicators of rapeseed response to fertilizer, it still has certain limitations. With respect to experimental design, this study employed a single-location, single-season design with only two fertilizer levels and did not measure baseline soil physicochemical properties (such as pH, organic matter, and total nitrogen), which limited the assessment of whether fertilizer was excessive or deficient. The sample size was limited (the correlation analysis was based on four varieties, *n* = 4), resulting in relatively low statistical power. At the molecular level, the cross-variety qPCR validation did not include biological replicates, nor did it systematically report primer amplification efficiency, include NTC/NRT controls, or verify the expression stability of the internal reference gene BnActin under different conditions. These limitations may affect the precise interpretation of the quantitative results. Therefore, the discussion of fertilizer application suitability and the conclusions regarding the cross-variety expression of candidate genes presented in this paper should be regarded as preliminary inferences, and the causal relationships between the candidate genes and yield and quality remain to be confirmed experimentally.

Future research plans to establish multi-level fertilizer application gradients across multiple sites and years, combined with baseline soil fertility data, to clarify the quantitative relationship between fertilizer application rates and yield and quality. In parallel, we plan to expand the number of varieties and biological replicates, refine the qPCR control system, and assess the stability of internal reference genes. Functional validation of the candidate genes will also be advanced through transgenic or gene-editing approaches. Finally, we intend to incorporate a comprehensive assessment of the economic benefits and the environmental effects of NPK fertilizer to further verify the stability and applicability of the aforementioned candidate indicators, thereby providing a more comprehensive scientific basis for rational fertilizer decisions in rapeseed production.

## 5. Conclusions

This study demonstrates that the growth-promoting effect of increased fertilizer on rapeseed is variety-dependent. Among the four varieties selected based on yield performance, CAT activity and TBARS content in stems at the initial flowering stage showed a positive correlation with yield. During the silique stage, the association between the *BnaC05g02370D* and *BnaC09g49910D* genes in stems and yield varied in direction under different fertilizer treatments. These physiological indicators and candidate genes may serve as promising markers for assessing rapeseed yield potential and provide a reference for the early screening of cultivation practices aimed at achieving high oil content and high yield.

## Figures and Tables

**Table 1:** ANOVA results for different rapeseed varieties under two fertilizer treatments at the bolting stage.

Agronomic Trait	Variety		Fertilizer		Variety × Fertilizer	
	F	*p*	F	*p*	F	*p*
Number of green leaves on the main stem	1.879	0.024	43.584	<0.001	5.343	<0.001
Total number of leaves on the main stem	2.398	0.003	42.667	<0.001	6.770	<0.001
Maximum leaf length	2.929	<0.001	41.687	<0.001	4.099	<0.001
Maximum leaf width	2.403	0.003	0.111	0.739	2.089	0.010
Root collar diameter	2.902	<0.001	51.304	<0.001	3.479	<0.001
Plant height	8.350	<0.001	7.215	0.008	7.976	<0.001

Note: Variety and fertilizer treatment were treated as fixed effects. The F values were derived from a two-way analysis of variance model that tested the two main effects of variety and fertilization and their interaction. The error term was provided by the variation among the five plants within each treatment combination, as five plants were sampled from each plot as replicates. Because each treatment combination corresponded to a single plot and no plot replication was available, the model did not incorporate a block random effect and was not fitted as a randomized complete block mixed model. Therefore, the analysis of variance and multiple comparisons should be regarded as preliminary. Normality was tested using the Kolmogorov–Smirnov test, with *n* = 180 (>50). For some traits, the residual variances did not satisfy the homogeneity assumption (Levene’s test, *p* < 0.05) or the normality assumption; however, the analysis of variance is reasonably robust to violations of these assumptions, so the results remain informative. *p* < 0.05 indicates a significant effect, and *p* < 0.01 indicates a highly significant effect. This note applies to the tables below.

**Table 2:** ANOVA results for different rapeseed varieties under two fertilizer treatments at the harvest stage.

Agronomic Trait	Variety		Fertilizer		Variety × Fertilizer	
	F	*p*	F	*p*	F	*p*
Plant height	8.176	<0.001	151.262	<0.001	2.361	0.003
Primary effective branch height	10.144	<0.001	6.127	0.014	3.388	<0.001
Effective length of main raceme	3.625	<0.001	8.134	0.005	1.983	0.016
No. of effective branches	4.772	<0.001	26.6	<0.001	2.294	0.004
No. of effective pods per plant	3.23	<0.001	59.6	<0.001	2.061	0.011
Root collar diameter	8.517	<0.001	0.824	0.366	3.014	<0.001
Yield per plant	2.113	0.009	50.125	<0.001	1.414	0.138

**Table 3:** Transcriptome sequencing quality assessment.

Sample	Raw Reads	Clean Reads	Effective Rate (%)	Raw Bases (G)	Raw GC (%)	Raw Q20 (%)	Raw Q30 (%)	Total_Mapped (%)	Unique (%)
NF_1	43,982,826	39,503,138	89.81	6.6	46.98	98.26	95.84	97.92	94.14
NF_2	42,943,478	38,909,838	90.61	6.44	46.96	98.36	96.07	98.12	94.08
NF_3	43,232,660	38,932,136	90.05	6.48	47.15	98.28	95.98	97.98	93.91
HF_1	44,070,264	39,552,288	89.75	6.61	47.09	98.22	95.82	98.04	93.9
HF_2	39,626,620	35,560,190	89.74	5.94	47.16	98.23	95.83	98.22	94.45
HF_3	41,897,262	37,541,870	89.6	6.28	47.21	98.22	95.83	98.2	94.27

Note: Raw reads denotes the number of raw sequencing reads; clean reads denotes the number of reads after quality control; effective rate refers to the percentage of clean reads relative to raw reads; Q20 and Q30 refer to the percentages of bases with quality scores ≥ 20 and ≥ 30, respectively; total_mapped refers to the percentage of clean reads mapped to the reference genome; unique refers to the percentage mapped to a unique position.

**Table 4:** Correlation analysis between pre-winter traits and yield in rapeseed.

Stage	Agronomic Trait Indicators	Index	
		HF	NF
Bolting Stage	Number of green leaves on the main stem	−0.623	0.536
	Total number of leaves on the main stem	−0.762	0.665
	Maximum leaf length	−0.254	0.645
	Maximum leaf width	0.947	0.961 *
	Root collar diameter	−0.110	−0.275
	Plant height	−0.535	0.788

Note: The correlation analysis was based on the mean values of the four varieties (*n* = 4). Asterisks denote significance levels, where * indicates *p* < 0.05 and ** indicates *p* < 0.01 (two-tailed test). The above correlation coefficients and their significance levels are provided only as a reference for exploratory trends, and the asterisks represent only the nominal significance level; therefore, they should not be used for robust statistical inference. The same notation applies to the following table.

**Table 5:** Correlation analysis of physiological index contents of rapeseed and yield.

Plant Tissues	Stage	Index			
		Superoxide Dismutase	Peroxidase	Catalase	Thiobarbituric Acid-Reactive Substances
Leaf	3–4 leaf stage	−0.189	−0.189	0.903 **	−0.476
	Bolting stage	0.043	0.043	0.594	0.579
	Initial flowering stage	0.054	0.594	0.325	−0.516
Stem	Initial flowering stage	0.632	−0.456	0.738 *	0.894 **
	Full flowering stage	−0.244	0.832 *	0.753 *	0.680
	Silique stage	0.771 *	0.681	−0.109	0.728 *

**Table 6:** Correlation analysis between rapeseed gene expression levels and yield.

Gene	Index	
	HF	NF
IAA17	−0.858	0.443
PYL5	0.977 *	0.806
IPT3	−0.933	−0.853
PR1	0.466	0.927

## Data Availability

The raw RNA-seq data generated in this study have been deposited in the NCBI Sequence Read Archive (SRA) under the BioProject accession number PRJNA1509745 and will be publicly available on 8 August 2026. The data can be accessed via https://www.ncbi.nlm.nih.gov/sra/PRJNA1509745, accessed on 8 August 2026.

## Supplementary Materials

The following supporting information can be downloaded at: https://www.mdpi.com/article/10.3390/life16081351/s1, The supplementary data include one PDF document (Supplementary Document.pdf) containing ten supplementary tables (Tables S1–S10) and six supplementary figures (Figures S1–S6) and one Excel file (Supplementary_Table_S11–S13.xlsx) containing three sheets (Normalized expression matrix, DEG list, and Metadata). The detailed titles are as follows: Table S1. Sampling schedule and measured traits across developmental stages; Table S2. Data coverage and scope of conclusions for each analysis; Table S3. Description and definitions of agronomic traits; Table S4. RT-qPCR gene primer sequences; Table S5. Agronomic traits during the pre-winter seedling stage under different fertilizer rates; Table S6. Agronomic traits of rapeseed at maturity under different fertilizer rates; Table S7. Quality indicators of rapeseed under different fertilizer rates; Figure S1. Scatter plots of SOD activity and yield per plant at different growth stages; (b) leaf at bolting stage; (c) leaf at initial flowering stage; (d) stem at initial flowering stage; (e) stem at full bloom stage; (f) stem at silique stage. Each dot represents one variety (n = 8). Red: HF; blue: NF. SOD activity is expressed as U·g^−1^·FW; Figure S2. Scatter plots of TBARS content and yield per plant at different growth stages. (a) Leaf at 3- to 4-leaf stage; (b) leaf at bolting stage; (c) leaf at initial flowering stage; (d) stem at initial flowering stage; (e) stem at full bloom stage; (f) stem at silique stage. Each dot represents one variety (n = 8). Red: HF; blue: NF. MDA content is expressed as μmol MDA eq.·g^−1^·FW; Figure S3. Scatter plots of POD activity and yield per plant at different growth stages. (a) Leaf at 3- to 4-leaf stage; (b) leaf at bolting stage; (c) leaf at initial flowering stage; (d) stem at initial flowering stage; (e) stem at full bloom stage; (f) stem at silique stage. Each dot represents one variety (n = 8). Red: HF; blue: NF. POD activity is expressed as U·g^−1^·min^−1^ FW; Figure S4. Scatter plots of CAT activity and yield per plant at different growth stages. (a) Leaf at 3- to 4-leaf stage; (b) leaf at bolting stage; (c) leaf at initial flowering stage; (d) stem at initial flowering stage; (e) stem at full bloom stage; (f) stem at silique stage. Each dot represents one variety (n = 8). Red: HF; blue: NF. CAT activity is expressed as U·g^−1^·min^−1^ FW; Figure S5A. Scatter plots of agronomic traits at bolting stage and yield per plant under NF treatment. (a) Number of green leaves on the main stem (No.); (b) total number of leaves on the main stem (No.); (c) maximum leaf length (cm); (d) maximum leaf width (cm); (e) root collar diameter (cm); (f) plant height (cm). Each dot represents one variety (n = 4); Figure S5B. Scatter plots of agronomic traits at bolting stage and yield per plant under HF treatment. (a) Number of green leaves on the main stem (No.); (b) total number of leaves on the main stem (No.); (c) maximum leaf length (cm); (d) maximum leaf width (cm); (e) root collar diameter (cm); (f) plant height (cm). Each dot represents one variety (n = 4); Figure S6A. Scatter plots of relative expression levels of candidate genes and yield per plant under NF treatment. (a) IAA17; (b) PYL5; (c) IPT3; (d) PR1. Each dot represents one variety (n = 4). Relative expression levels were normalized to the internal control; Figure S6B. Scatter plots of relative expression levels of candidate genes and yield per plant under HF treatment. (a) IAA17; (b) PYL5; (c) IPT3; (d) PR1. Each dot represents one variety (n = 4). Relative expression levels were normalized to the internal control; Table S8. Pearson correlation coefficients between physiological indices and yield per plant in leaf and stem at different growth stages in rapeseed; Table S9A. Correlations between agronomic traits at bolting stage and yield per plant under NF treatment; Table S9B. Correlations between agronomic traits at bolting stage and yield per plant under HF treatment; Table S10A. Correlations between relative expression levels of candidate genes and yield per plant under NF treatment; Table S10B. Correlations between relative expression levels of candidate genes and yield per plant under HF treatment; Table S11. Normalized expression matrix; Table S12. DEG list; Table S13. Metadata.

## Abbreviations

The following abbreviations are used in this manuscript:

HF	High fertilizer
NF	Normal fertilizer
RT-qPCR	Real-time quantitative polymerase chain reaction
ROS	Reactive oxygen species
