# Supplementary material for: Effects of Fertilizer Rates on Growth of Brassica napus L
Source: Life (Basel). 2026 Aug 17;16(8):1351. doi: 10.3390/life16081351 (PMC13514223; doi:10.3390/life16081351)
Supplement: Supplementary file 1 [file life-16-01351-s001.zip › life-4413723-Supplementary Document.pdf]

Table S1. Sampling schedule and measured traits across developmental stages.

Calendar date	Growth stage	Tissue	Trait	Number of Biological Replicates	Trial/Yea
2023-11-01	3- to 4-leaf stage	Leaf	Physiological indicators (SOD, CAT, POD, TBARS)	3	2023
2024-01-12	Bolting stage	Leaf	Physiological indicators (SOD, CAT, POD, TBARS) and Pre-winter seedling-stage indicators	3	2024
2024-02-05	Initial flowering stage	Leaf and Stem	Physiological indicators (SOD, CAT, POD, TBARS)	3	2024
2024-02-15	Full bloom stage	Stem	Physiological indicators (SOD, CAT, POD, TBARS)	3	2024
2024-04-09	Silique stage	Stem	Physiological indicators (SOD, CAT, POD, TBARS) and transcriptome	3,1 (candidate gene qPCR validation)	2024
2024-05-05	Harvest time	Seeds	Harvest traits and quality traits	3,1 (quality traits)	2024

Note: The experiment included two fertilization levels (normal fertilization, NF; high fertilization, HF) and 18 varieties, with each treatment combination corresponding to a single subplot and no subplot-level (spatial) replicates. For within-variety comparisons of physiological indicators, four varieties—Z01, Z02, Huayouza 50, and Huayouza 652—were selected. Trends between treatments were described using mean values; no significance tests were conducted, and no significance letters were assigned. Owing to the limited sample size, nonparametric tests for both NF and HF revealed no significant differences ($P > 0.05$). A two-factor analysis of variance (ANOVA) was performed for each agronomic trait, with variety and fertilization as the two factors. As each treatment combination consisted of a single plot without spatial replicates, the error term was derived mainly from the variation among the five plants within each combination; consequently, the ANOVA results and related statistics should be interpreted only as indicative of general trends. The qPCR analysis of candidate genes comprised two parts. First, to verify the reliability of the transcriptomic sequencing data, qPCR was performed on candidate genes using RNA samples from variety Z01 under the NF and HF treatments, with three independent biological replicates per treatment and three technical replicates per biological replicate. Second, to examine the expression trends of the candidate genes across the four varieties, qPCR was conducted using a single RNA sample per variety per treatment, with three technical replicates per sample, because the remaining RNA after field sampling was insufficient to support three biological replicates per variety. The results were used to assess the expression trends of the candidate genes. Quality traits were determined using mixed seed samples.

Table S2. Data coverage and scope of conclusions for each analysis.

Analysis procedure	Data used	Sample scope	Applicability of conclusions
Variety screening	Yield per plant of 18 varieties × 2 treatments	18 varieties → selection of 4: Z01, Z02, Huayouza 50, Huayouza 652	—
Quality analysis (oil content, fatty acids, etc.)	18 varieties × 2 treatments × no replicates	18 varieties	Conclusions apply to the 18 varieties
Pre-winter traits and harvest stage traits	18 varieties × 2 treatments × 5 replicates	18 varieties	Conclusions apply to the 18 varieties
Physiological indicators (SOD, CAT, POD, TBARS)	4 varieties × 2 treatments × 3 replicates	4 varieties (Z01, Z02, Huayouza 50, Huayouza 652)	Conclusions apply to the 4 tested varieties
Transcriptome sequencing and differential gene screening	Z01 × 2 treatments × 3 replicates，Pearson correlation analysis	Only Z01	Conclusions apply only to variety Z01
Candidate gene qPCR validation	Based on Z01 transcriptome screening, 4 varieties × 2 treatments × no replicates	4 varieties	qPCR validation results apply to the 4 tested varieties

Table S3. Description and definitions of agronomic traits.

Trait	Definition	Unit
Number of green leaves on the main stem	Number of green functional leaves on the main stem	No.
Total number of leaves on the main stem	Total emerged leaves on the main stem (including green leaves and leaf scars)	No.
Maximum leaf length	Length of the largest leaf on the main stem, measured from leaf base to tip	cm
Maximum leaf width	Maximum width of the largest leaf on the main stem	cm
Plant height	Vertical distance from the cotyledonary node to the main stem apex	cm
Primary effective branch height	Distance from the cotyledonary node to the lowest primary effective branch insertion point	cm
Effective length of main raceme	Length of the main raceme from the uppermost to the lowermost effective pod-bearing node	cm
No. of effective branches	Number of primary branches bearing at least one effective silique	No.
No. of effective pods per plant	Total number of effective siliques per plant	No.
Root collar diameter	Diameter at the root–stem junction below the cotyledonary node	cm
Yield per plant	Average seed yield per plant from yield component assessment samples	g

Table S4. RT-qPCR gene primer sequence.

Gene ID	Name	Upstream primers	Downstream primers
BnActin	BnActin	CGTTGGTGGAGTTGCACTTG	AGCACGTTACGGGATTGGTT
BnaC08g09640D	IAA2	TCTCCCACCTTGTTCTTCCTTT	AGGGGACAAAGATGGGACAC
BnaA10g02490D	IAA3	GCCAAAGGTTTAGGATGCGG	AGGTTCCAGTTTCATGTGCT
BnaA03g36950D	IAA7	AAGATGGCGACTGGATGCTC	GAACCCGTCATATGGTTGAACA
BnaC05g02370D	IAA17	TTCCACCTTCCTCATCACCTT	CCCATCATTGTGAACCTCTAGTT
BnaA06g02010D	IAA18	AAAGACCCACTACTCCACCTCT	TTCACGCAACAGAAACTCTCA
BnaC08g19010D	SAUR40	TGAATCGGGTGGCCAGTTAC	AGCCCTTTGAAATCAACTCACT
BnaC09g49910D	PYL5	CGGTGCAACCTTCAGTCTCT	CCCTCCAACAATATTTTGATTCCCA
BnaC04g01830D	NPR5	CGAGTCGTTAGCACTGCAC	CACCGAGTTCAAGCAAAGCC
BnaC03g45470D	PR1	ACGTGCATTAATGGAGAATGCC	ACCTTGCTTTGCCACATCCA
BnaA04g00130D	IPT3	TCAAGACTGCGATGAGTGGAG	ACAAGGCGAGCAAGTCTCAT
BnaA08g24500D	NRT2.5	CTCGGCTAATGGTATCGCC	CCGAACAAGAGAACGGCAAAA

TableS5. Agronomic traits during the pre-winter seedling stage under different fertilizer rates.

Materials	Treatment	Number of green leaves on the main stem (No.)	Total number of leaves on the main stem (No.)	Maximum leaf length (cm)	Maximum leaf width (cm)	Root collar diameter (cm)	Plant height (cm)

1	NF1	8.20±1.30b	9.60±1.52b	20.80±1.92b	13.00±1.46a	1.86±0.35a	13.20±1.64b
	HF1	11.00±1.41a	12.20±1.48a	24.92±2.06a	14.84±1.84a	1.28±0.13b	20.88±2.28a
2	NF2	9.20±2.28a	10.80±0.84a	21.60±2.70a	14.20±1.30a	1.82±0.13a	15.20±1.79b
	HF2	9.20±1.10a	11.20±1.30a	22.22±2.13a	13.90±1.42a	1.24±0.13b	20.56±1.46a
3	NF3	7.60±0.55a	8.60±0.55a	23.20±1.30a	14.60±1.67a	2.26±0.23a	13.00±2.74a
	HF3	7.60±0.55a	9.40±0.89a	22.22±2.13a	14.52±1.50a	1.52±0.30b	11.94±1.26a
4	NF4	8.20±0.84a	9.80±0.84a	21.60±2.30a	14.20±1.64a	2.12±0.16a	26.60±6.11a
	HF4	6.60±1.34a	7.20±1.30b	21.24±1.07a	11.52±2.20b	1.68±0.59b	17.80±3.68b
5	NF5	9.40±2.30a	10.40±2.70a	19.40±3.78a	15.06±3.19a	2.06±0.68a	16.80±5.67a
	HF5	6.80±1.10b	7.80±1.30b	19.42±4.88a	11.76±2.18b	1.66±0.36a	10.94±2.59b
6	NF6	8.60±1.14a	10.20±0.84a	20.20±3.90a	15.20±3.70a	2.16±0.36a	21.20±4.09a
	HF6	7.40±1.34a	8.00±1.22b	18.54±1.72a	12.82±1.47b	1.54±0.41b	20.26±5.08a
7	NF7	9.20±0.45a	10.20±0.45a	21.00±3.81a	16.00±2.24a	2.06±0.56a	16.80±3.56a
	HF7	9.60±0.55a	11.00±0.71a	17.52±1.25a	13.64±1.04a	1.34±0.05b	12.40±1.02b
8	NF8	6.80±0.84b	8.20±0.45b	17.42±1.95b	13.80±1.02a	1.72±0.33a	14.82±2.16a
	HF8	10.40±2.07a	12.20±2.17a	20.80±1.25a	13.82±1.84a	1.62±0.20a	18.10±1.14a
9	NF9	9.00±1.58a	10.20±1.48a	19.16±3.20a	13.50±2.77a	1.80±0.60a	18.78±3.43a
	HF9	7.80±1.92a	8.80±1.92a	22.32±3.06a	13.82±1.62a	1.08±0.11b	14.56±0.75b
10	NF10	6.60±0.89b	8.40±1.14a	19.98±2.14a	14.44±1.83a	1.90±0.35a	16.76±3.93a
	HF10	9.80±0.45a	11.60±0.89b	19.08±1.26a	14.82±1.55a	1.22±0.08b	14.70±0.84a
11	NF11	7.00±0.71b	8.20±0.84b	19.26±2.98b	15.20±1.99a	1.90±0.32a	16.88±2.19a
	HF11	9.00±0.10a	10.20±0.84a	22.70±2.59a	16.88±1.64a	1.20±0.10b	17.94±0.43a
12	NF12	7.60±1.14b	8.80±0.84b	19.74±1.65b	13.96±0.62a	1.46±0.38a	15.58±1.88a
	HF12	9.80±1.10a	11.60±1.52a	25.40±2.07a	15.52±0.79a	1.24±0.09a	16.60±0.74a
13	NF13	7.60±0.55b	8.60±0.55a	20.04±1.74a	15.14±2.11a	1.84±0.30a	14.64±2.29a
	HF13	9.80±1.10a	10.80±1.64b	23.02±1.85a	15.30±0.57a	1.32±0.08b	15.70±1.04a
14	NF14	7.00±0.71a	8.80±1.10a	17.92±2.22b	13.82±2.48a	1.50±0.51a	10.30±2.53b
	HF14	8.60±2.30a	9.80±2.17a	22.58±2.14a	15.22±2.61a	1.22±0.04a	14.00±1.12a
15	NF15	6.80±1.64b	7.80±1.64b	16.88±2.07b	13.02±1.43a	1.42±0.46a	16.42±2.42b
	HF15	10.20±1.30a	12.00±1.58a	23.90±2.70a	12.74±1.37a	1.32±0.15a	21.40±0.82a
16	NF16	7.00±0.10b	7.60±0.55b	18.08±2.51b	13.10±1.58a	1.78±0.38a	14.46±3.08b
	HF16	10.20±2.39a	11.80±1.48a	25.10±2.39a	15.10±1.58a	1.46±0.11a	20.40±1.47a
17	NF17	7.20±2.68b	8.60±1.52b	17.20±2.03b	11.28±1.47a	1.38±0.26b	14.30±1.31a
	HF17	10.20±0.84a	11.00±1.00a	20.60±2.07a	13.00±1.58a	1.90±0.22a	17.60±3.36a
18	NF18	7.20±1.64b	8.60±1.95b	18.38±4.37a	12.40±2.49a	1.52±0.47b	12.60±1.57b
	HF18	10.60±1.34a	10.80±1.10a	20.60±2.88a	14.40±1.82a	2.16±0.42a	22.60±4.34a

Note: The correspondence between material codes and varieties in the table is as follows: 1, Zhongshuang 11; 2, Z01; 3, Z02; 4, Z03; 5, Zhongyou 814; 6, Zhongyou 926; 7, Zheyou 505; 8, Zhongyou 827; 9, Zhongyou Hybrid 652; 10, Huayou Hybrid 50; 11, Huayou Hybrid 652; 12, Fengyou 789; 13, Zheyou Hybrid 59; 14, Zheyou Hybrid 313; 15, Fengyou 958; 16, Fengyou 823; 17, Fengyou 919; 18, Shengyuan No. 1. A two-way factorial ANOVA and simple effects analysis were used. Different lowercase letters in the same column indicate significant differences between NF and HF treatments for

the same variety at the P < 0.05 level; the same applies to the tables below.

TableS6. Agronomic traits of rapeseed at maturity under different fertilizer rates.

Materials	Treatment	Plant height (cm)	Primary effective branch height (cm)	Effective length of main raceme (cm)	No. of effective branches （No.）	No. of effective pods per plant （No.）	Root collar diameter(cm)	Yield per plant (g)
1	NF1	139.20±3.96b	49.80±1.30a	55.40±4.83a	8.60±1.67a	227.60±89.26b	2.04±0.22a	11.69±4.41b
	HF1	150.80±7.16a	52.20±6.34a	57.60±3.29a	9.20±2.17a	364.80±124.09a	2.24±0.29a	18.12±7.20a
2	NF2	135.60±5.13b	58.80±9.04a	58.80±4.02b	6.00±1.00b	88.40±7.57b	1.64±0.11b	9.02±0.29b
	HF2	163.20±5.67a	53.80±7.16a	70.40±5.68a	9.40±1.14a	316.20±111.99a	1.98±0.27a	17.76±6.99a
3	NF3	144.80±3.11b	48.80±6.65a	58.80±4.97a	7.40±0.89b	244.20±32.51b	1.70±0.19a	9.80±1.82b
	HF3	160.60±8.35a	47.40±8.11a	63.40±3.21a	9.60±2.70a	354.80±143.18a	1.86±0.17a	18.22±7.16a
4	NF4	154.60±1.95b	77.00±8.63a	59.00±10.12a	5.60±1.14a	187.20±62.77a	1.80±0.16a	11.48±3.29a
	HF4	173.40±6.07a	80.00±8.43a	52.8±25.82a	5.60±0.55a	241.2±69.72a	1.70±0.43a	16.95±6.43a
5	NF5	137.60±5.98b	59.00±12.21a	51.00±12.33a	7.60±2.07a	221.00±84.73b	1.74±0.15a	9.13±3.52b
	HF5	153.00±9.14a	61.60±17.43a	54.00±12.65a	8.00±2.83a	333.40±153.7a	1.68±0.31a	16.31±7.40a
6	NF6	135.80±4.09b	44.80±8.23a	54.40±6.11a	7.80±0.84a	201.40±58.54a	1.68±0.13a	15.7±2.50a
	HF6	152.20±9.50a	52.40±10.95a	64.40±8.20a	7.40±0.89a	255.20±47.62a	1.44±0.26a	13.09±2.90a
7	NF7	155.40±6.19a	74.40±10.01a	55.80±4.27a	6.00±1.22a	175.20±91.41a	1.54±0.26a	12.25±4.63a
	HF7	157.00±6.00a	68.60±8.32a	55.20±3.27a	6.60±1.14a	235.20±98.27a	1.84±0.17a	16.09±5.89a
8	NF8	140.60±4.04b	59.40±6.88a	53.20±4.49b	6.60±1.14a	149.40±45.07a	1.46±0.17a	9.83±2.08a
	HF8	159.40±10.60a	59.40±12.07a	65.40±4.04a	8.00±1.00a	243.60±75.26a	1.28±0.30a	15.18±3.73a
9	NF9	111.00±24.38b	47.40±3.13b	28.00±18.33b	4.60±1.67b	91.80±24.41b	1.46±0.23a	4.20±2.29b
	HF9	146.80±11.78a	63.60±17.21a	50.60±3.44a	8.40±1.34a	248.80±47.13a	1.34±0.15a	12.17±1.90a
10	NF10	141.80±9.76b	61.00±8.66a	51.60±11.55a	7.20±1.30a	165.80±43.16b	1.68±0.04a	8.00±1.50b
	HF10	162.60±13.96a	67.40±13.92a	58.80±4.09a	8.60±1.95a	293.60±104.83a	1.66±0.26a	17.30±5.39a
11	NF11	140.20±6.38a	46.80±8.87a	65.60±5.22a	7.20±1.79b	189.00±31.47a	1.46±0.21a	10.76±2.70b
	HF11	150.60±14.62a	42.00±18.67a	55.60±7.20a	10.40±2.19a	244.00±115.22a	1.62±0.48a	19.76±14.04a
12	NF12	133.00±12.37b	40.80±7.85b	51.60±6.15a	9.00±1.00a	247.20±50.75a	1.46±0.26a	11.42±1.66a
	HF12	159.60±13.78a	59.20±7.66a	57.40±10.33a	10.00±1.22a	268.60±60.87a	1.66±0.18a	13.88±6.31a
13	NF13	149.20±8.35b	52.60±6.43b	58.40±0.55a	8.20±0.84a	232.60±49.35b	2.08±0.19a	14.72±3.32b
	HF13	168.20±8.26a	66.40±8.62a	54.60±5.13a	9.00±1.00a	361.20±15.85a	1.94±0.13a	22.02±5.88a
14	NF14	147.00±8.37b	48.80±10.5b	55.00±7.42a	8.8±1.92a	286.80±104.09a	2.28±0.55a	16.02±6.39a
	HF14	163.40±3.51a	72.80±13.18a	49.80±4.97a	8.00±1.41a	286.40±66.63a	1.76±0.22b	13.20±3.51a
15	NF15	129.20±9.04b	54.60±9.89a	53.20±6.38a	5.80±0.84a	158.00±24.24a	1.24±0.11a	5.76±0.80b
	HF15	154.20±4.21a	64.80±6.14a	60.20±4.32a	6.20±1.10a	244.00±96.22a	1.28±0.22a	14.43±6.44a
16	NF16	136.80±4.55b	35.80±5.54a	58.60±2.88a	8.60±1.34a	322.60±30.84a	1.66±0.11a	14.38±1.69a
	HF16	148.60±9.32a	48.20±15.39a	56.80±7.79a	7.80±2.95a	282.60±112.91a	1.50±0.38a	15.67±6.49a
17	NF17	145.20±8.38a	57.00±6.82a	57.80±12.52a	6.60±0.89b	176.00±54.15b	1.34±0.13b	8.57±1.84a
	HF17	148.60±5.77a	29.60±12.18b	62.60±7.23a	10.40±2.70a	427.80±163.24a	2.04±0.22a	11.69±4.41a
18	NF18	158.40±11.10a	73.40±9.91a	56.00±14.35a	7.00±0.00a	169.60±25.44a	1.68±0.15a	11.43±2.03a
	HF18	163.00±4.69a	68.00±5.83a	59.60±4.39a	7.60±0.89a	213.00±21.68a	1.72±0.20a	16.82±2.62a

TableS7. Quality indicators of rapeseed under different fertilizer rates.

Treatment	Saturated fatty acid （%）		Unsaturated fatty acid（%）			Oil content （%）	Thioglycoside （%）	Protein （%）
	Palmitic acid（%）	Stearic acid（%）	Oleic acid（%）	Linoleic acid（%）	Linolenic acid（%）			
NF1	4.97	2.04	62.42	20.47	8.69	43.31	20.61	20.33
HF1	4.74	2.17	65.16	19.67	8.56	41.22	19.26	23.55
NF2	3.82	2.47	72.19	16.17	7.71	47.88	19.78	21.61
HF2	3.96	2.61	73.69	15.91	7.05	46.04	24.13	23.46
NF3	4.16	2.39	74.22	18.29	8.36	51.91	27.64	18.69
HF3	4.60	2.38	66.92	20.11	8.35	46.23	23.61	22.59
NF4	3.98	2.48	69.82	17.14	8.42	47.80	21.39	21.92
HF4	4.03	2.10	72.13	15.79	8.37	43.55	24.85	25.19
NF5	4.08	2.21	61.75	15.41	6.89	44.51	37.24	20.71
HF5	4.32	2.61	70.55	15.53	4.85	40.87	36.54	22.47
NF6	4.48	2.43	70.09	18.60	6.79	44.13	32.73	20.52
HF6	4.69	2.32	66.87	19.32	7.74	40.59	23.96	24.91
NF7	4.57	2.11	65.16	19.76	9.92	46.68	20.75	21.04
HF7	4.61	2.18	67.14	19.52	9.07	43.48	25.05	23.29
NF8	4.29	2.59	70.93	18.79	8.20	49.12	22.41	19.72
HF8	4.50	2.26	70.79	19.19	8.37	42.01	25.84	25.60

NF9	4.61	2.63	63.44	20.33	8.61	42.20	25.89	23.94
HF9	4.60	2.37	69.38	19.67	8.20	43.50	23.27	23.46
NF10	4.40	2.53	64.45	17.68	7.90	44.61	27.18	20.59
HF10	4.19	2.03	73.74	16.90	8.36	43.38	19.75	25.07
NF11	4.66	2.31	63.39	18.89	7.56	42.49	28.11	20.94
HF11	4.64	2.28	67.22	18.65	7.30	41.08	27.47	22.64
NF12	4.50	2.37	66.59	18.58	7.66	45.71	39.27	19.01
HF12	4.66	2.13	66.56	18.75	7.72	42.62	28.88	21.82
NF13	4.33	2.32	63.98	18.07	8.60	45.07	24.05	21.25
HF13	4.14	2.36	71.95	16.59	7.50	43.08	22.44	22.83
NF14	4.24	2.38	68.27	17.71	8.50	49.23	17.04	18.22
HF14	4.48	2.43	65.73	19.54	7.92	44.88	23.97	20.71
NF15	4.50	2.48	63.75	17.74	7.21	44.30	33.25	19.93
HF15	4.35	2.27	68.94	16.84	7.32	44.94	29.84	20.79
NF16	4.34	2.28	63.10	17.62	7.64	44.30	41.81	19.75
HF16	4.56	2.48	63.83	18.32	7.35	42.60	42.07	21.22
NF17	4.55	2.25	63.09	18.23	7.89	43.90	31.33	20.32
HF17	4.41	2.22	62.97	18.40	7.91	42.57	27.97	22.79
NF18	4.33	2.44	75.32	17.77	7.42	46.86	13.66	20.56
HF18	4.11	2.22	71.57	17.26	8.09	45.20	20.08	23.01

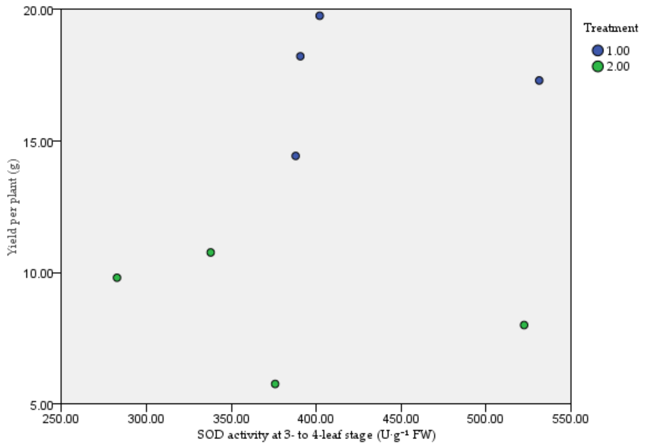
Treatment
1.00
2.00
Yield per plant (g)
20.00
15.00
10.00
5.00
250.00
300.00
350.00
400.00
450.00
500.00
550.00
SOD activity at 3- to 4-leaf stage (U·g⁻¹ FW)

(a)

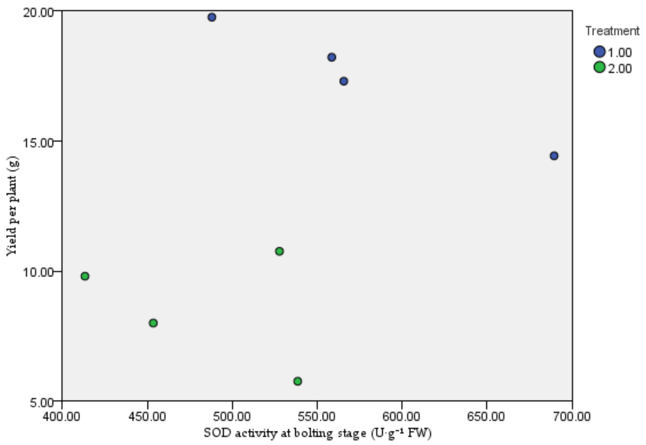
Treatment
1.00
2.00
Yield per plant (g)
20.00
15.00
10.00
5.00
400.00
450.00
500.00
550.00
600.00
650.00
700.00
SOD activity at bolting stage (U·g⁻¹ FW)

(b)

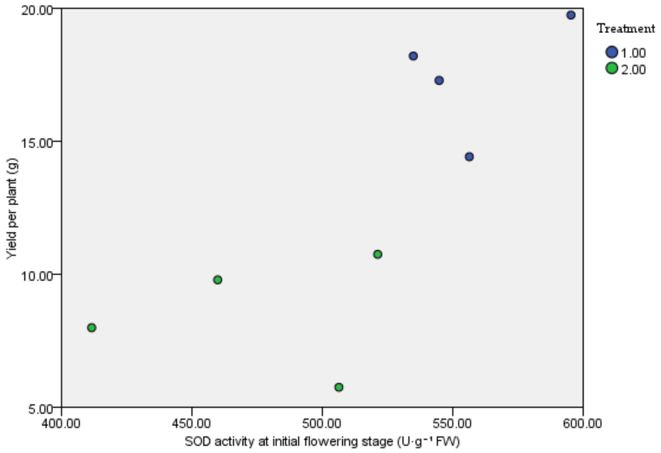
Treatment
1.00
2.00
Yield per plant (g)
20.00
15.00
10.00
5.00
400.00
450.00
500.00
550.00
600.00
SOD activity at initial flowering stage (U·g⁻¹ FW)

(c)

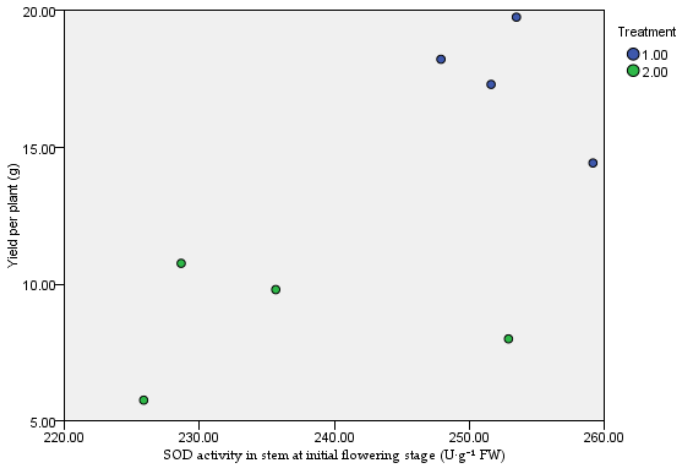

(d)

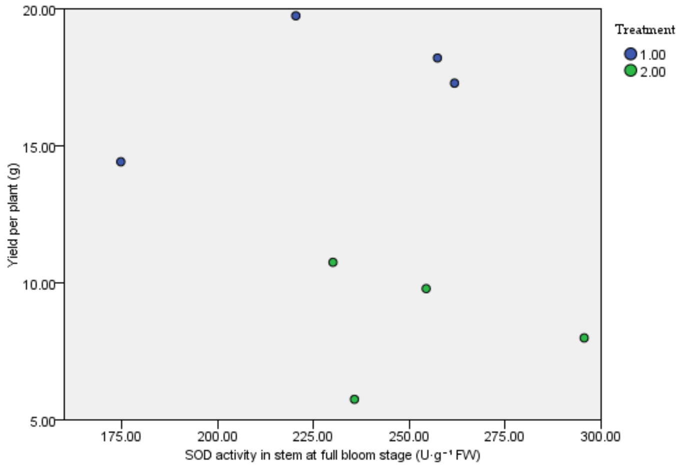

(e)

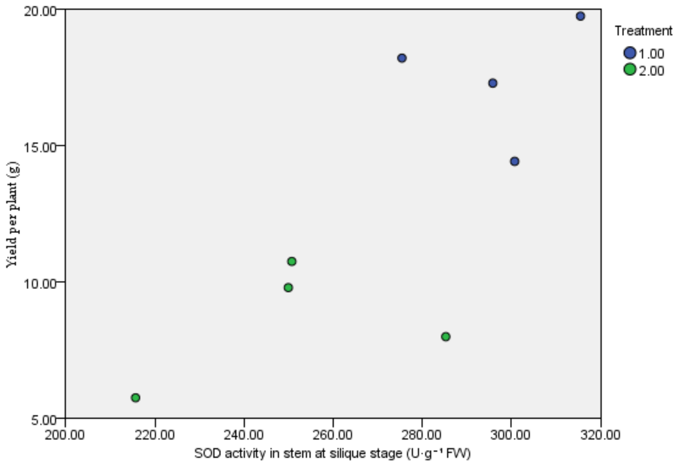

(f)

Figure S1. Scatter plots of SOD activity and yield per plant at different growth stages.(a) Leaf at 3- to 4-leaf stage; (b) leaf at bolting stage; (c) leaf at initial flowering stage; (d) stem at initial flowering stage;

(e) stem at full bloom stage; (f) stem at silique stage. Each dot represents one variety (n=8). Red: HF; blue: NF. SOD activity is expressed as U·g^{-1}·FW.

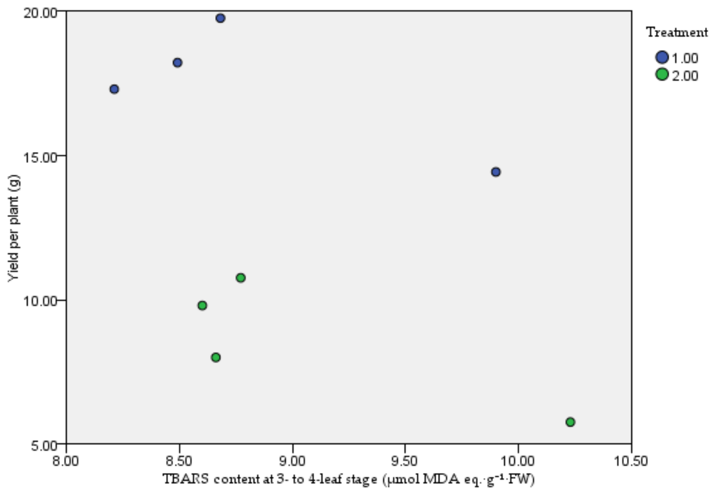

(a)

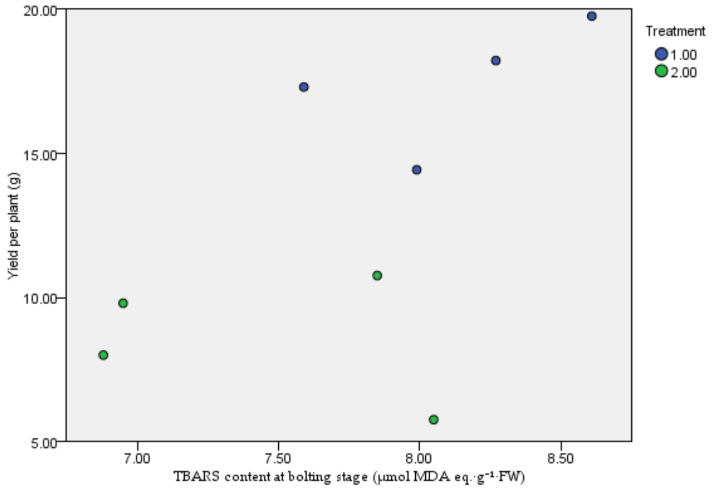

(b)

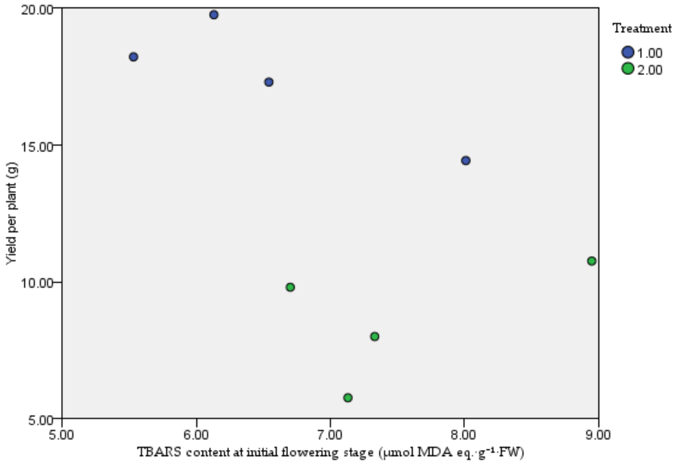
20.00
15.00
10.00
5.00
5.00
6.00
7.00
8.00
9.00
Yield per plant (g)
TBARS content at initial flowering stage (μmol MDA eq.·g⁻¹·FW)
Treatment
1.00
2.00

(c)

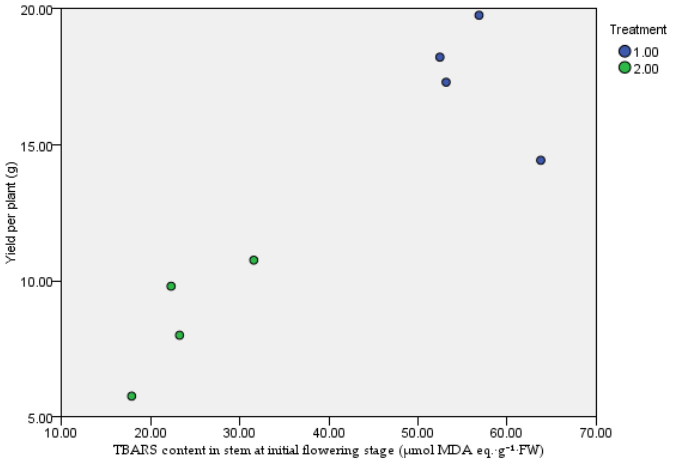
20.00
15.00
10.00
5.00
10.00
20.00
30.00
40.00
50.00
60.00
70.00
Yield per plant (g)
TBARS content in stem at initial flowering stage (μmol MDA eq.·g⁻¹·FW)
Treatment
1.00
2.00

(d)

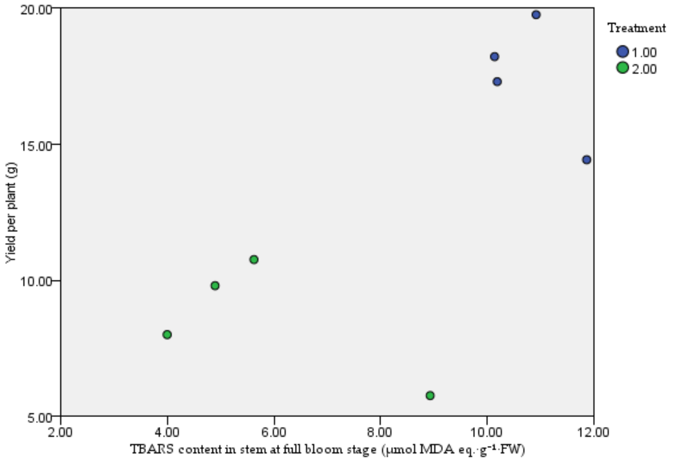
20.00
15.00
10.00
5.00
2.00
4.00
6.00
8.00
10.00
12.00
Yield per plant (g)
TBARS content in stem at full bloom stage (μmol MDA eq.·g⁻¹·FW)
Treatment
1.00
2.00

(e)

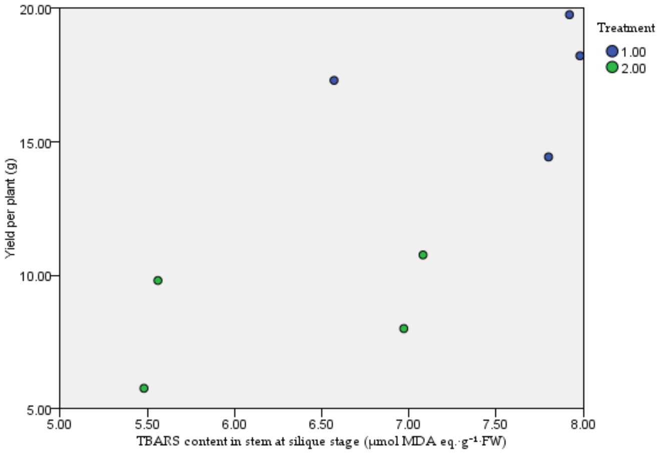

(f)

Figure S2. Scatter plots of TBARS content and yield per plant at different growth stages. (a) Leaf at 3- to 4-leaf stage; (b) leaf at bolting stage; (c) leaf at initial flowering stage; (d) stem at initial flowering stage; (e) stem at full bloom stage; (f) stem at silique stage. Each dot represents one variety (n=8). Red: HF; blue: NF. MDA content is expressed as μmol MDA eq.·g^{-1}·FW.

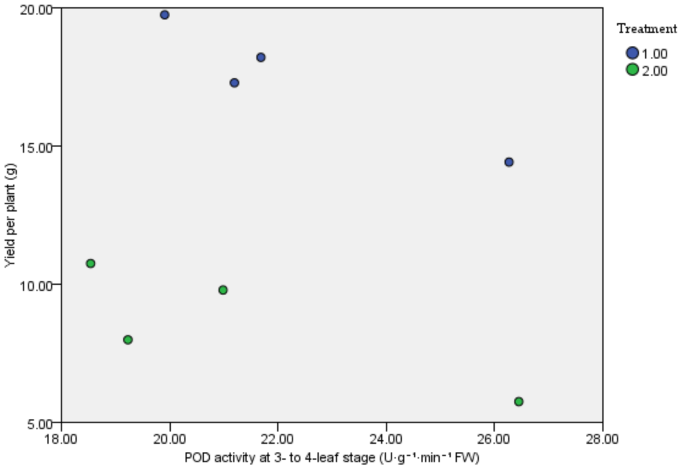

(a)

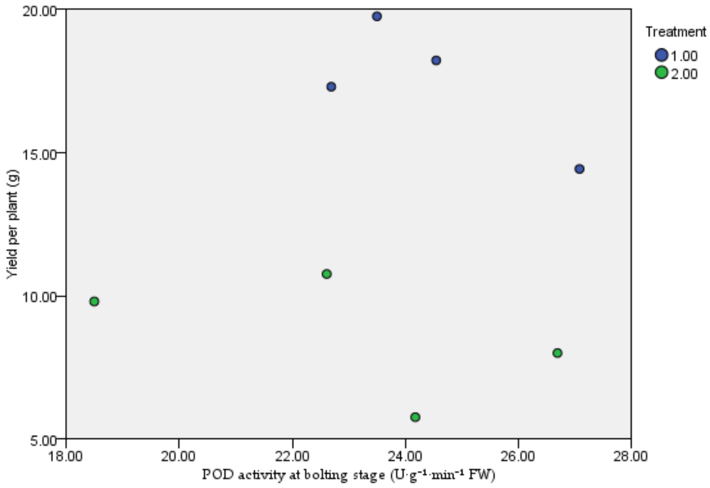
Treatment
1.00
2.00
Yield per plant (g)
POD activity at bolting stage (U·g⁻¹·min⁻¹ FW)
20.00
15.00
10.00
5.00
18.00
20.00
22.00
24.00
26.00
28.00

(b)

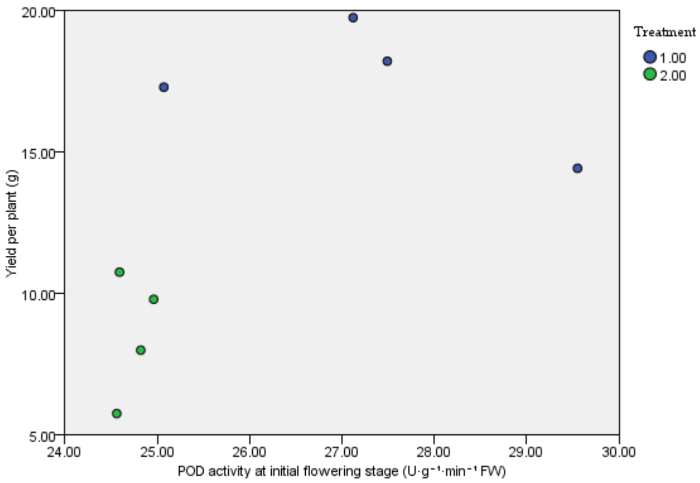
Treatment
1.00
2.00
Yield per plant (g)
POD activity at initial flowering stage (U·g⁻¹·min⁻¹ FW)
20.00
15.00
10.00
5.00
24.00
25.00
26.00
27.00
28.00
29.00
30.00

(c)

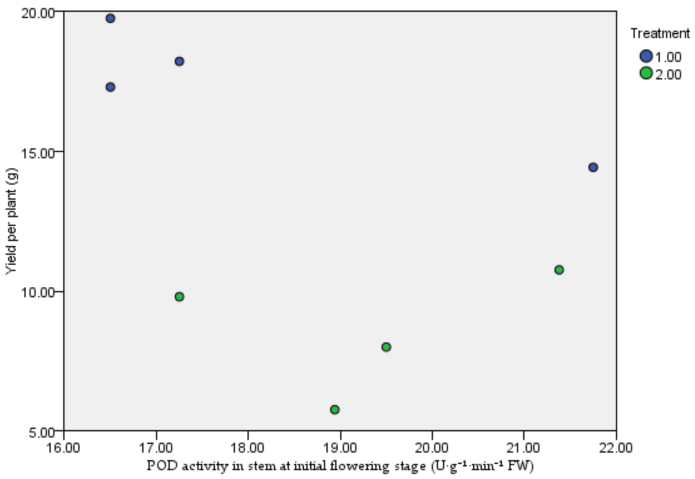
Treatment
1.00
2.00
Yield per plant (g)
POD activity in stem at initial flowering stage (U·g⁻¹·min⁻¹ FW)
20.00
15.00
10.00
5.00
16.00
17.00
18.00
19.00
20.00
21.00
22.00

(d)

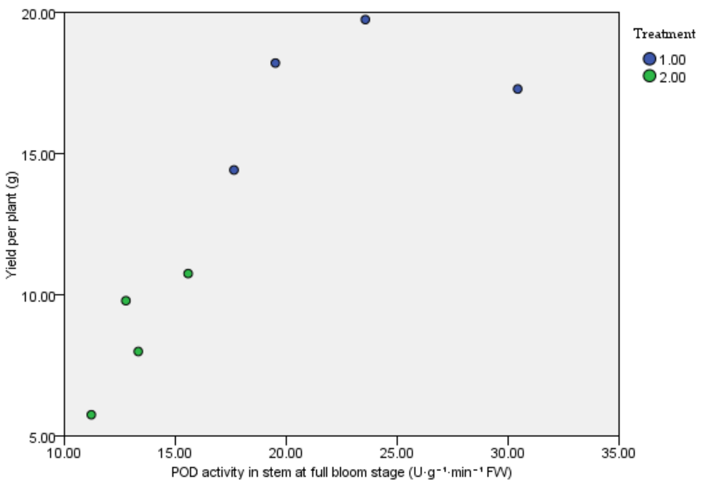

(e)

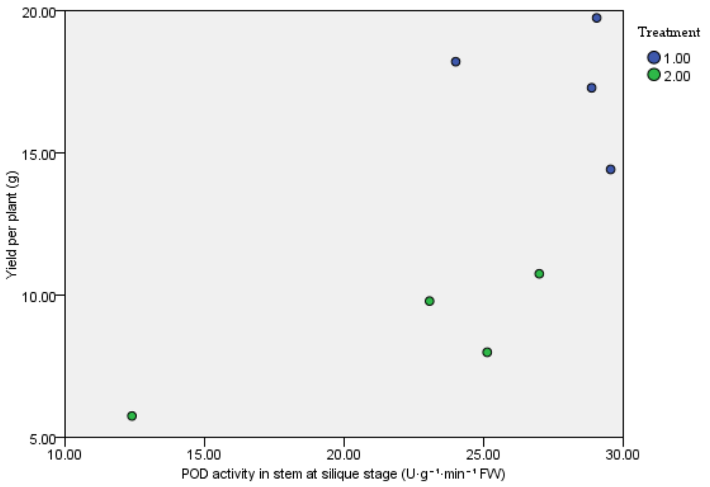

(f)

Figure S3. Scatter plots of POD activity and yield per plant at different growth stages. (a) Leaf at 3- to 4-leaf stage; (b) leaf at bolting stage; (c) leaf at initial flowering stage; (d) stem at initial flowering stage; (e) stem at full bloom stage; (f) stem at silique stage. Each dot represents one variety (n=8). Red: HF; blue: NF. POD activity is expressed as $U{\cdot}g^{-1}{\cdot}min^{-1}$ FW.

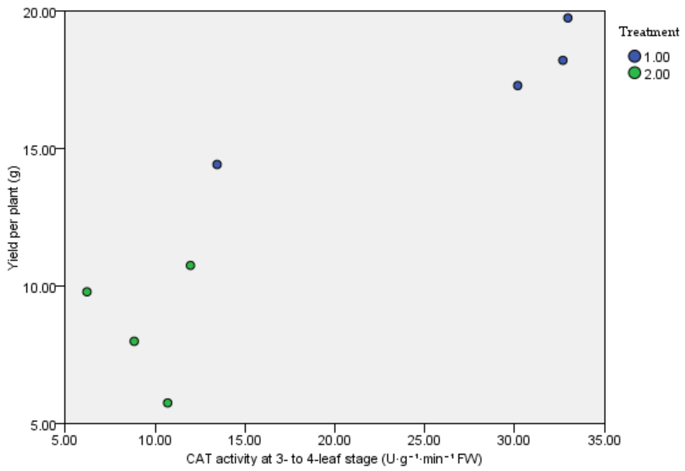
Treatment
1.00
2.00
Yield per plant (g)
CAT activity at 3- to 4-leaf stage (U·g⁻¹·min⁻¹ FW)
20.00
15.00
10.00
5.00
5.00
10.00
15.00
20.00
25.00
30.00
35.00

(a)

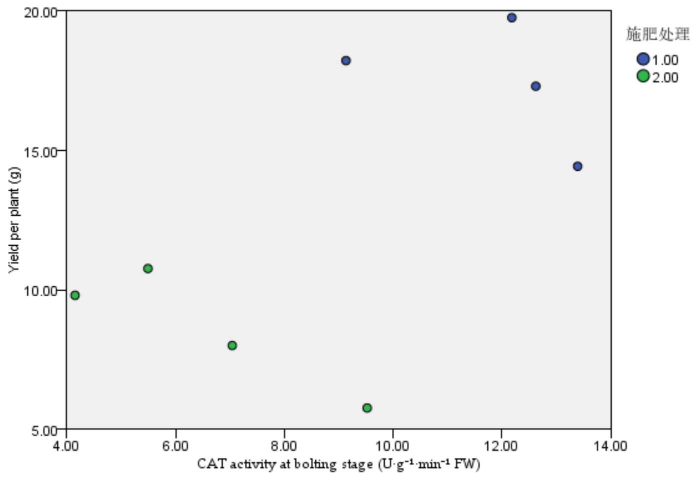
施肥处理
1.00
2.00
Yield per plant (g)
CAT activity at bolting stage (U·g⁻¹·min⁻¹ FW)
20.00
15.00
10.00
5.00
4.00
6.00
8.00
10.00
12.00
14.00

(b)

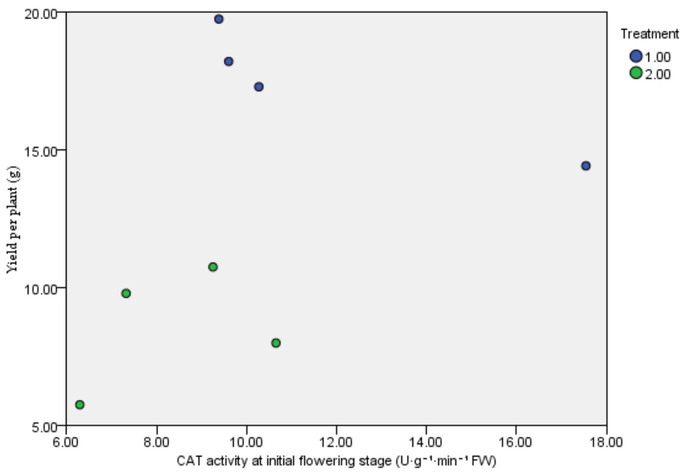
Treatment
1.00
2.00
Yield per plant (g)
CAT activity at initial flowering stage (U·g⁻¹·min⁻¹ FW)
20.00
15.00
10.00
5.00
6.00
8.00
10.00
12.00
14.00
16.00
18.00

(c)

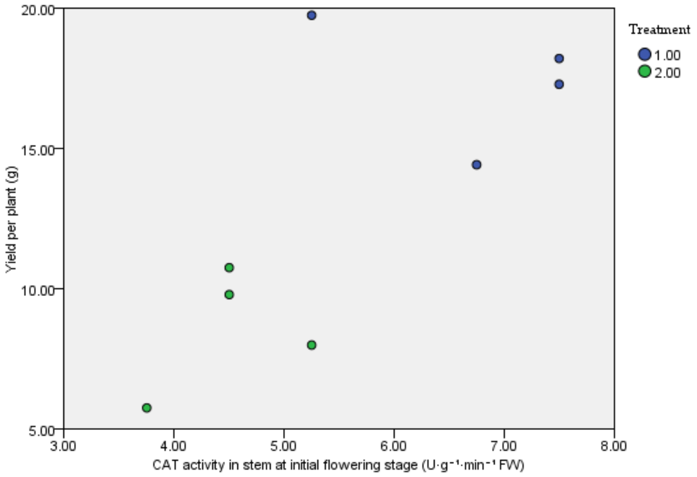
Treatment
1.00
2.00
Yield per plant (g)
CAT activity in stem at initial flowering stage (U·g⁻¹·min⁻¹ FW)

(d)

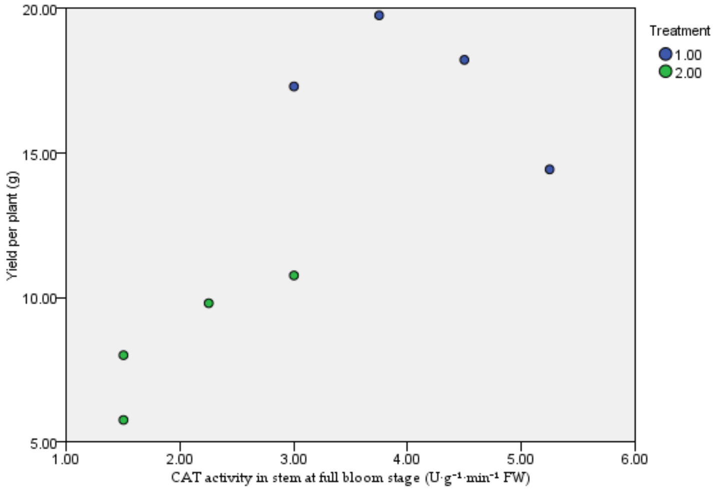
Treatment
1.00
2.00
Yield per plant (g)
CAT activity in stem at full bloom stage (U·g⁻¹·min⁻¹ FW)

(e)

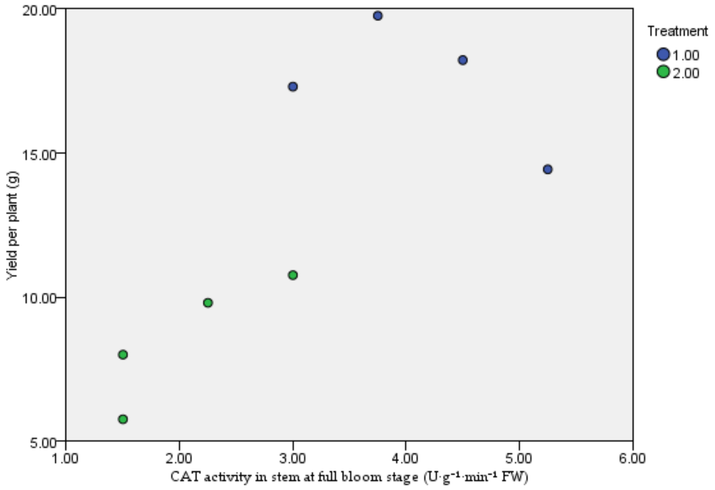
Treatment
1.00
2.00
Yield per plant (g)
CAT activity in stem at full bloom stage (U·g⁻¹·min⁻¹ FW)

(f)

Figure S4. Scatter plots of CAT activity and yield per plant at different growth stages. (a) Leaf at 3- to 4-leaf stage; (b) leaf at bolting stage; (c) leaf at initial flowering stage; (d) stem at initial flowering stage; (e) stem at full bloom stage; (f) stem at silique stage. Each dot represents one variety (n=8). Red: HF; blue: NF. CAT activity is expressed as U·g⁻¹·min⁻¹ FW.

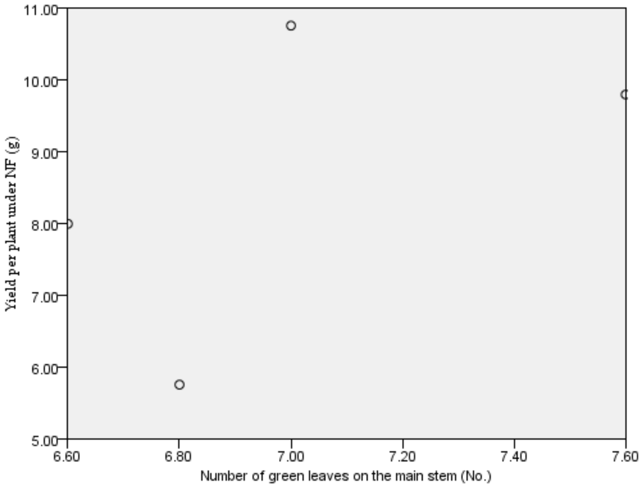

(a)

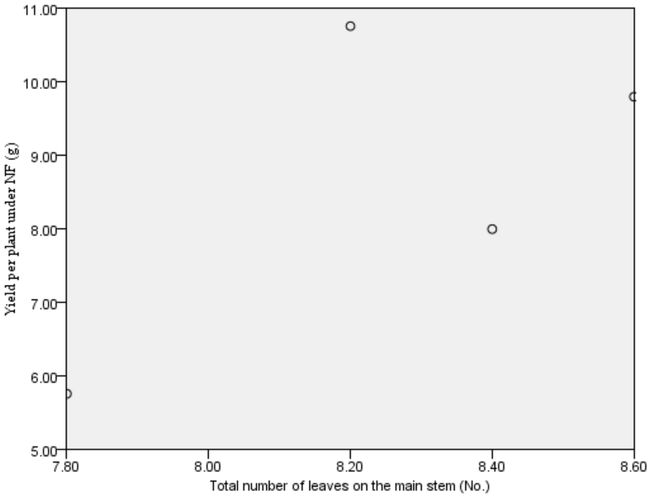

(b)

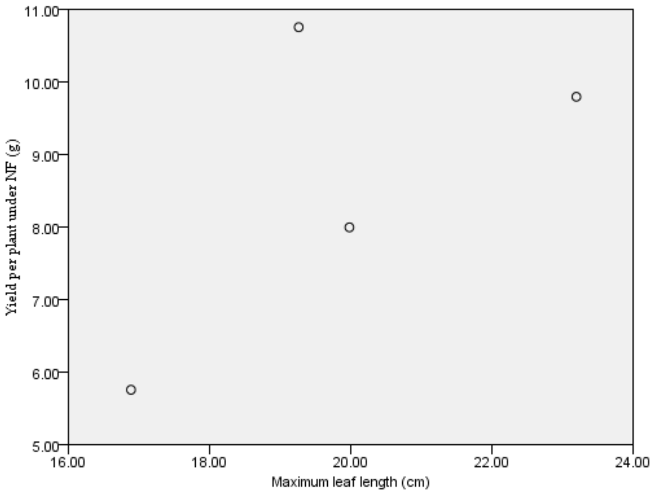

Yield per plant under NF (g)
11.00
10.00
9.00
8.00
7.00
6.00
5.00
16.00
18.00
20.00
22.00
24.00
Maximum leaf length (cm)

(c)

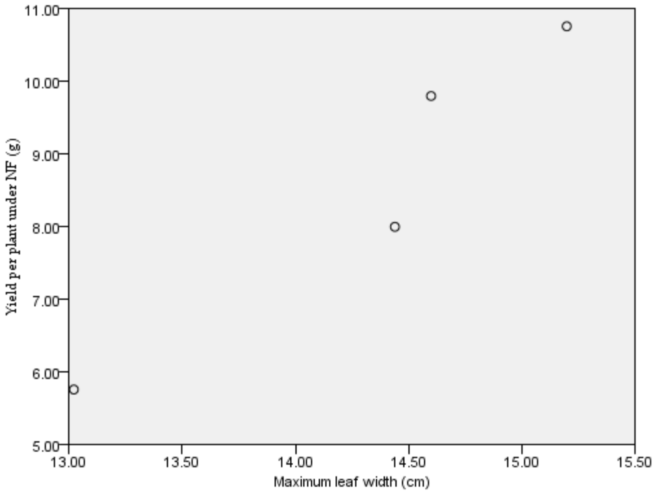

Yield per plant under NF (g)
11.00
10.00
9.00
8.00
7.00
6.00
5.00
13.00
13.50
14.00
14.50
15.00
15.50
Maximum leaf width (cm)

(d)

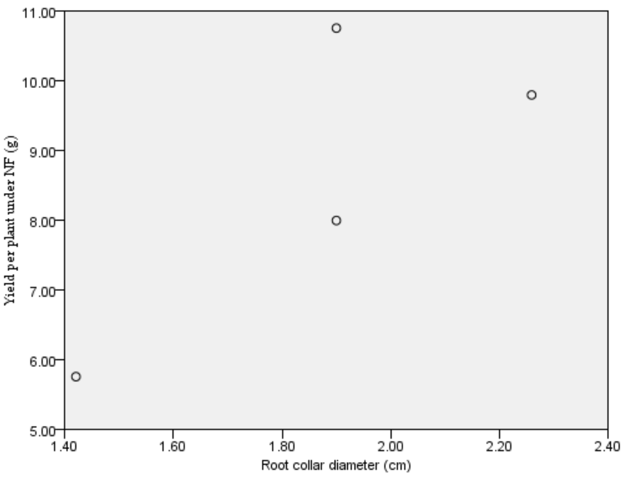

Yield per plant under NF (g)
11.00
10.00
9.00
8.00
7.00
6.00
5.00
1.40
1.60
1.80
2.00
2.20
2.40
Root collar diameter (cm)

(e)

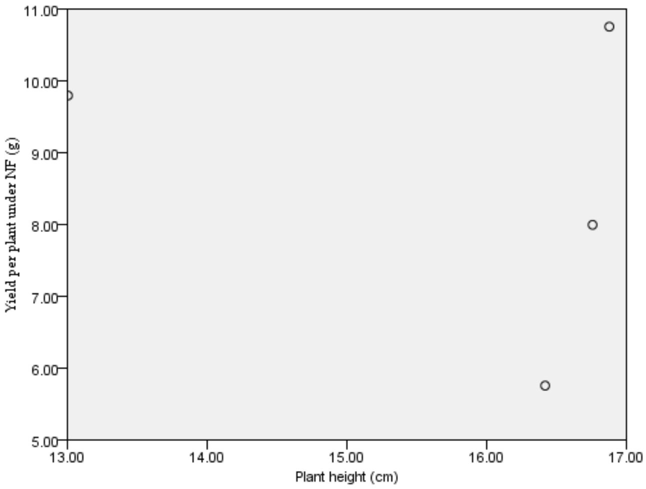

(f)

Figure S5A. Scatter plots of agronomic traits at bolting stage and yield per plant under NF treatment. (a) Number of green leaves on the main stem (No.); (b) total number of leaves on the main stem (No.); (c) maximum leaf length (cm); (d) maximum leaf width (cm); (e) root collar diameter (cm); (f) plant height (cm). Each dot represents one variety (n=4).

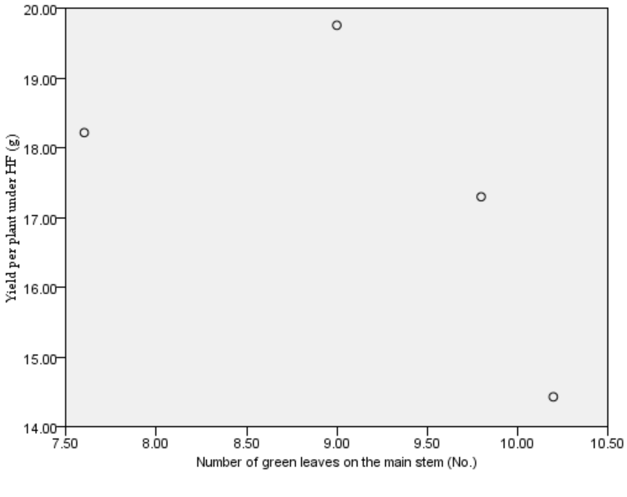

(a)

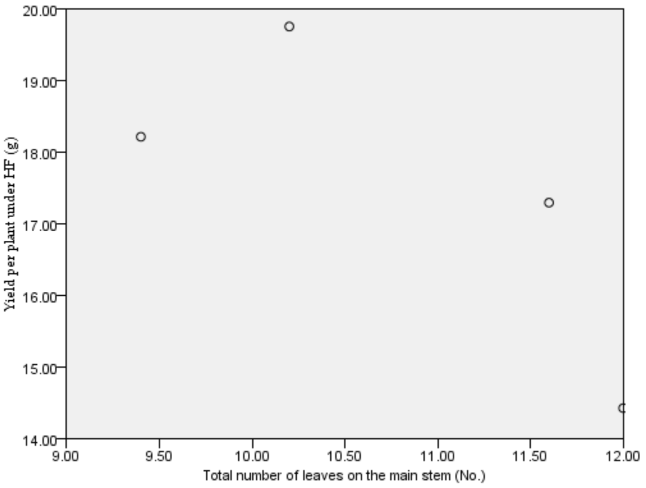

Yield per plant under HF (g)
Total number of leaves on the main stem (No.)

(b)

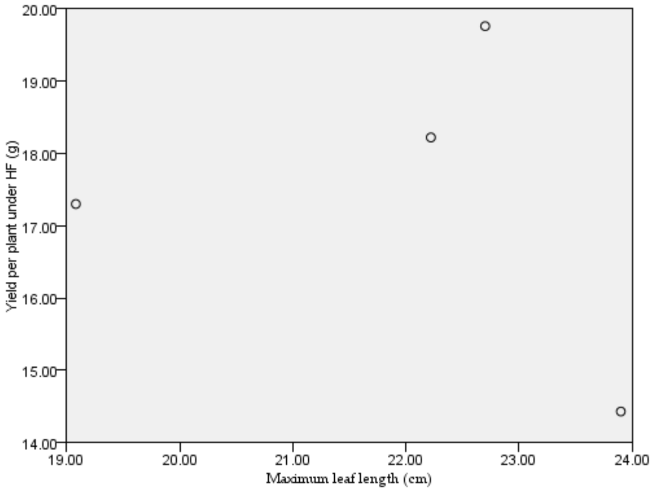

Yield per plant under HF (g)
Maximum leaf length (cm)

(c)

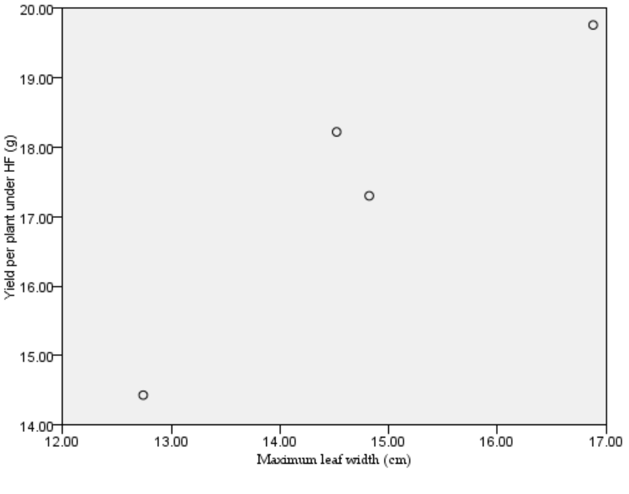

Yield per plant under HF (g)
Maximum leaf width (cm)

(d)

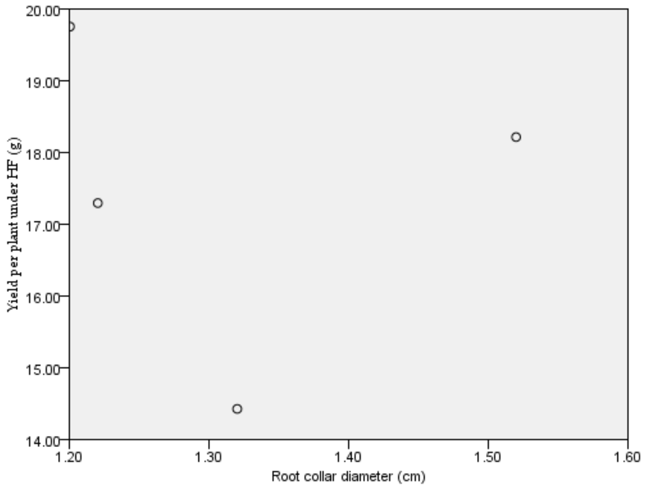

(e)

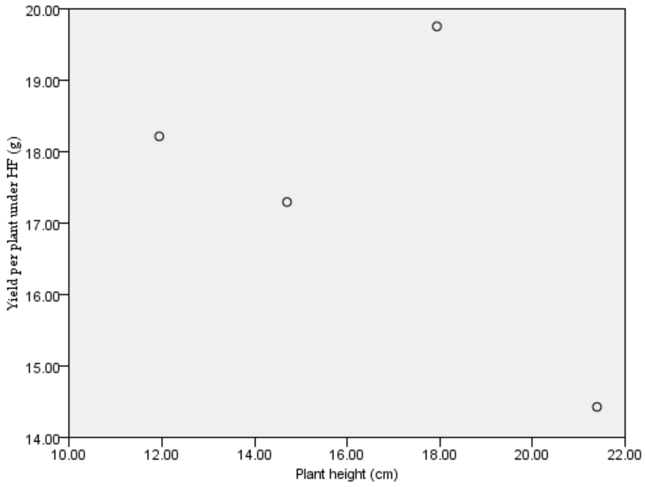

(f)

Figure S5B. Scatter plots of agronomic traits at bolting stage and yield per plant under HF treatment. (a) Number of green leaves on the main stem (No.); (b) total number of leaves on the main stem (No.); (c) maximum leaf length (cm); (d) maximum leaf width (cm); (e) root collar diameter (cm); (f) plant height (cm). Each dot represents one variety (n=4).

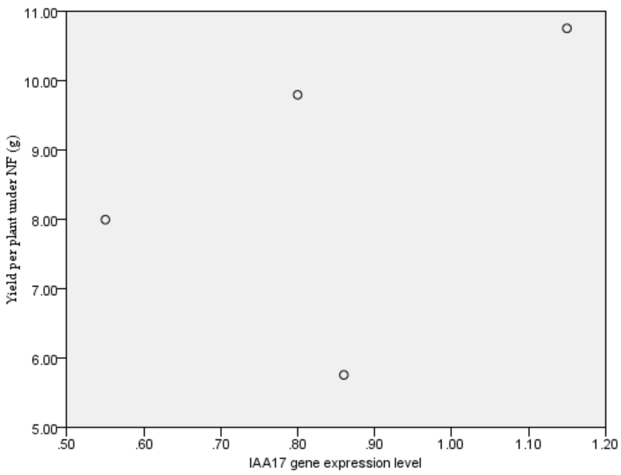
Yield per plant under NF (g)
IAA17 gene expression level

(a)

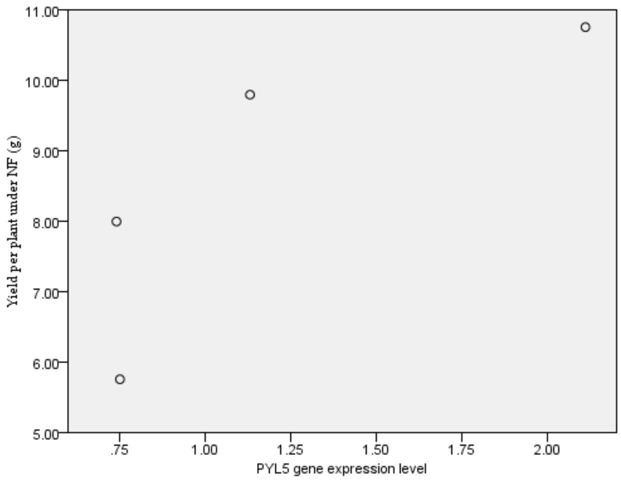
Yield per plant under NF (g)
PYL5 gene expression level

(b)

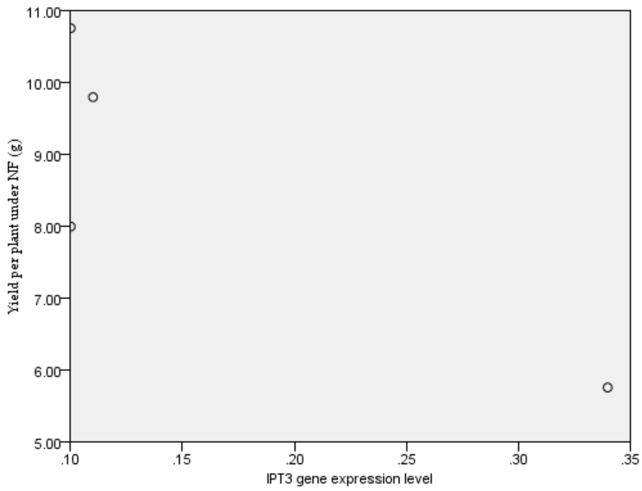
Yield per plant under NF (g)
IPT3 gene expression level

(c)

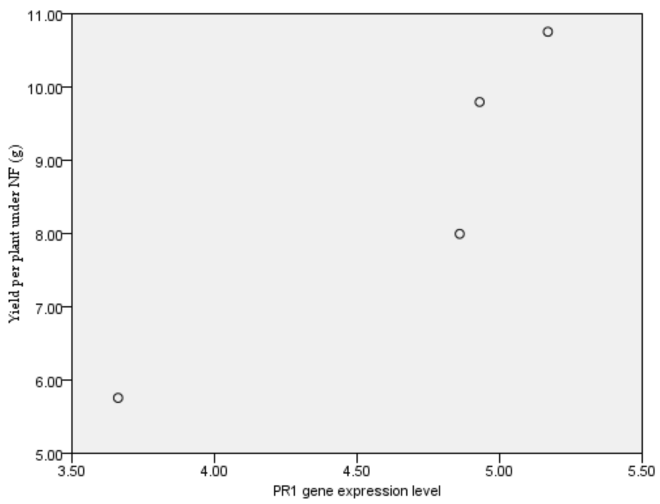

(d)

Figure S6A. Scatter plots of relative expression levels of candidate genes and yield per plant under NF treatment. (a) IAA17; (b) PYL5; (c) IPT3; (d) PR1. Each dot represents one variety (n=4). Relative expression levels were normalized to the internal control.

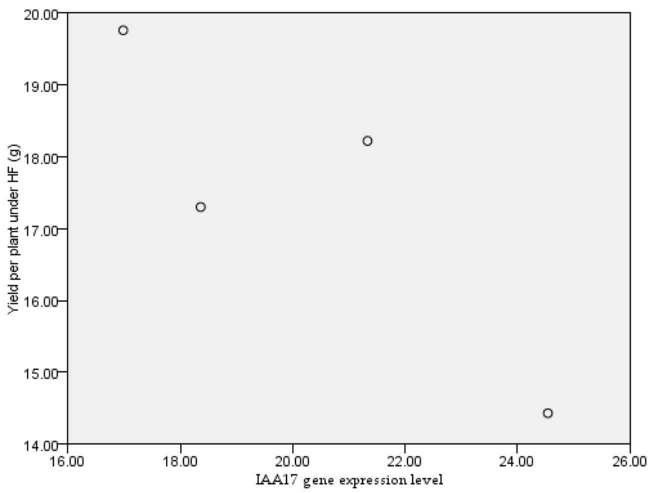

(a)

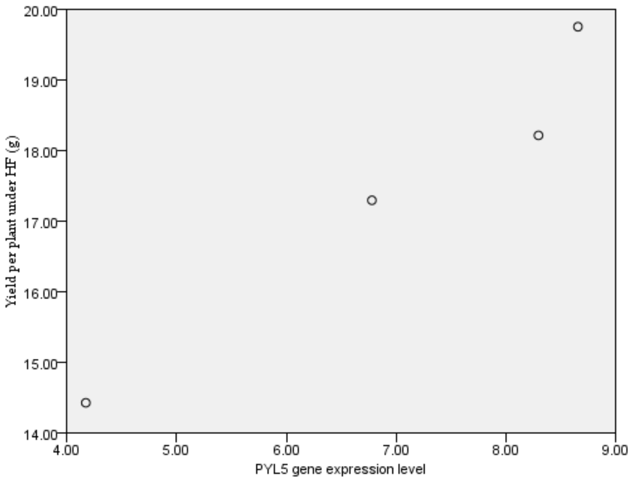

20.00
19.00
18.00
17.00
16.00
15.00
14.00
Yield per plant under HF (g)
4.00
5.00
6.00
7.00
8.00
9.00
PYL5 gene expression level

(b)

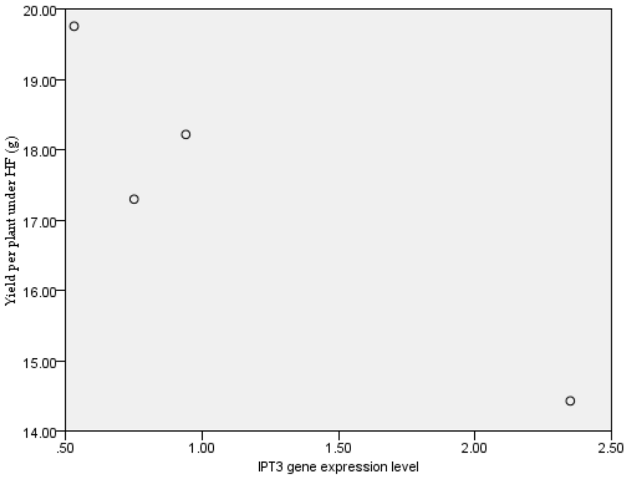

20.00
19.00
18.00
17.00
16.00
15.00
14.00
Yield per plant under HF (g)
.50
1.00
1.50
2.00
2.50
IPT3 gene expression level

(c)

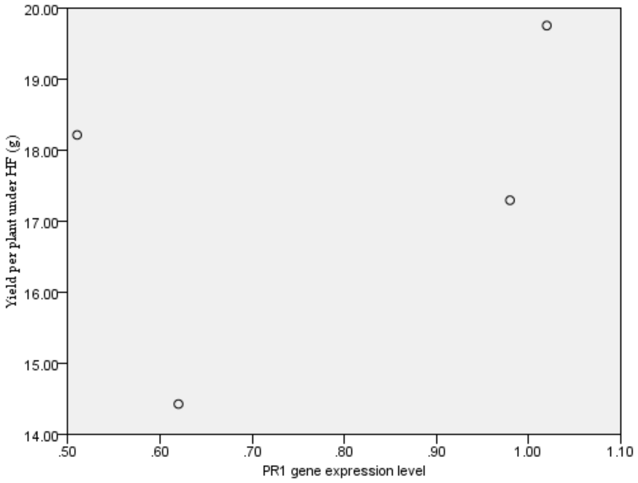

20.00
19.00
18.00
17.00
16.00
15.00
14.00
Yield per plant under HF (g)
.50
.60
.70
.80
.90
1.00
1.10
PR1 gene expression level

(d)

Figure S6B. Scatter plots of relative expression levels of candidate genes and yield per plant under HF treatment. (a) IAA17; (b) PYL5; (c) IPT3; (d) PR1. Each dot represents one variety (n=4). Relative expression levels were normalized to the internal control.

TableS8. Pearson correlation coefficients between physiological indices and yield per plant in leaf and stem at different growth stages in rapeseed.

Plant Tissues		Leaf			Stem		
Stage		3- to 4-leaf stage	Bolting Stage	Initial flowering stage	Initial flowering stage	Full bloom stage	Silique stage
Superoxide dismutase	r value	0.192	0.314	0.757	0.632	-0.244	0.771*
	p value	0.648	0.449	0.03	0.093	0.561	0.025
	95% CI	(-0.603, 0.818)	(-0.501, 0.859)	(0.108, 0.956)	(-0.133, 0.930)	(-0.831, 0.552)	(0.132, 0.960)
Peroxidase	r value	-0.189	0.043	0.594	-0.456	0.832*	0.681
	p value	0.655	0.919	0.121	0.256	0.01	0.063
	95% CI	(-0.808, 0.596)	(-0.679, 0.733)	(-0.164, 0.916)	(-0.898, 0.281)	(0.260, 0.974)	(-0.040, 0.944)
Catalase	r value	0.903**	0.594	0.325	0.738*	0.753*	-0.109
	p value	0.002	0.121	0.432	0.037	0.031	0.797
	95% CI	(0.501, 0.987)	(-0.164, 0.916)	(-0.496, 0.862)	(0.079, 0.952)	(0.097, 0.956)	(-0.770, 0.647)
Thiobarbituric acid reactive substances	r value	-0.476	0.579	-0.516	0.894**	0.68	0.728*
	p value	0.233	0.133	0.191	0.003	0.063	0.041
	95% CI	(-0.905, 0.241)	(-0.183, 0.912)	(-0.920, 0.184)	(0.468, 0.985)	(-0.041, 0.944)	(0.058, 0.950)

Note: *Values are Pearson correlation coefficients (r) with p values and 95% confidence intervals in parentheses. n = 8 for all correlation analyses. *p < 0.05, *p < 0.01.

Table S9A. Correlations between agronomic traits at bolting stage and yield per plant under NF treatment.

agronomic trait indicators	r value	p value	95% CI
Number of green leaves on the main stem	0.536	0.464	(-0.826, 0.990)
Total number of leaves on the main stem	0.665	0.335	(-0.661, 0.997)
Maximum leaf length	0.645	0.355	(-0.685, 0.997)
Maximum leaf width	0.961*	0.039	(0.189, 1.000)
Root collar diameter	0.788	0.212	(-0.447, 0.999)
Plant height	-0.275	0.725	(-0.985, 0.933)

Note: Values are Pearson correlation coefficients (r) with p values and 95% confidence intervals in parentheses. n = 4 for all correlation analyses. *$p < 0.05$. The same applies to the following tables.

Table S9B.Correlations between agronomic traits at bolting stage and yield per plant under HF treatment.

agronomic trait indicators	r value	p value	95% CI
Number of green leaves on the main stem	-0.623	0.377	(-0.995, 0.729)
Total number of leaves on the main stem	-0.762	0.238	(-0.999, 0.426)
Maximum leaf length	-0.254	0.746	(-0.983, 0.940)
Maximum leaf width	0.947	0.053	(-0.126, 1.000)
Root collar diameter	-0.11	0.89	(-0.966, 0.945)
Plant height	-0.535	0.465	(-0.992, 0.827)

Table S10A. Correlations between relative expression levels of candidate genes and yield per plant under NF treatment.

Gene	r value	p value	95% CI
IAA17	0.443	0.557	(-0.894, 0.993)
PYL5	0.806	0.194	(-0.378, 0.999)
IPT3	-0.853	0.147	(-0.999, 0.144)
PR1	0.927	0.073	(-0.490, 1.000)

Table S10B.Correlations between relative expression levels of candidate genes and yield per plant under HF treatment.

Gene	r value	p value	95% CI
IAA17	-0.858	0.142	(-0.999, 0.082)
PYL5	0.977*	0.023	(0.410, 1.000)
IPT3	-0.933	0.067	(-1.000, -0.263)
PR1	0.466	0.534	(-0.884, 0.994)
